# *In vivo *modeling of metastatic human high-grade serous ovarian cancer in mice

**DOI:** 10.1371/journal.pgen.1008808

**Published:** 2020-06-04

**Authors:** Olga Kim, Eun Young Park, David L. Klinkebiel, Svetlana D. Pack, Yong-Hyun Shin, Zied Abdullaev, Robert E. Emerson, Donna M. Coffey, Sun Young Kwon, Chad J. Creighton, Sanghoon Kwon, Edmund C. Chang, Theodore Chiang, Alexander N. Yatsenko, Jeremy Chien, Dong-Joo Cheon, Yang Yang-Hartwich, Harikrishna Nakshatri, Kenneth P. Nephew, Richard R. Behringer, Facundo M. Fernández, Chi-Heum Cho, Barbara Vanderhyden, Ronny Drapkin, Robert C. Bast, Kathy D. Miller, Adam R. Karpf, Jaeyeon Kim

**Affiliations:** 1 Department of Biochemistry and Molecular Biology, Indiana University Melvin and Bren Simon Comprehensive Cancer Center, Indiana University School of Medicine, Indianapolis, Indiana, United States of America; 2 Department of Biochemistry and Molecular Biology, Fred & Pamela Buffett Cancer Center, University of Nebraska Medical Center, Omaha, Nebraska, United States of America; 3 Laboratory of Pathology, Center for Cancer Research, National Cancer Institute, National Institutes of Health, Bethesda, Maryland, United States of America; 4 Department of Pathology and Laboratory Medicine, Indiana University School of Medicine, Indianapolis, Indiana, United States of America; 5 Department of Pathology and Genomic Medicine, Houston Methodist and Weill Cornell Medical College, Houston, Texas, United States of America; 6 Department of Pathology, School of Medicine, Keimyung University, Daegu, Republic of Korea; 7 Department of Medicine, Baylor College of Medicine, Houston, Texas, United States of America; 8 Research and Development Center, Bioway Inc, Seoul, Republic of Korea; 9 Human Genome Sequencing Center, Baylor College of Medicine, Houston, Texas, United States of America; 10 Department of Obstetrics, Gynecology & Reproductive Sciences, Magee-Womens Research Institute, University of Pittsburgh, Pittsburgh, Pennsylvania, United States of America; 11 Department of Biochemistry and Molecular Medicine, University of California, Davis, Sacramento, California, United States of America; 12 Department of Regenerative and Cancer Cell Biology, Albany Medical College, Albany, NY, United States of America; 13 Department of Obstetrics, Gynecology, and Reproductive Sciences, Yale School of Medicine, New Haven, Connecticut, United States of America; 14 Department of Surgery, Indiana University Melvin and Bren Simon Comprehensive Cancer Center, Indiana University School of Medicine, Indianapolis, Indiana, United States of America; 15 Medical Sciences Program, Indiana University Melvin and Bren Simon Comprehensive Cancer Center, Indiana University School of Medicine, Bloomington, Indiana, United States of America; 16 Departments of Genetics, University of Texas MD Anderson Cancer Center, Houston, Texas, United States of America; 17 School of Chemistry and Biochemistry, Georgia Institute of Technology, Atlanta, Georgia, United States of America; 18 Department of Obstetrics and Gynecology, School of Medicine, Keimyung University, Daegu, Republic of Korea; 19 Department of Cellular and Molecular Medicine, University of Ottawa, and Ottawa Hospital Research Institute, Ottawa, Ontario, Canada; 20 Penn Ovarian Cancer Research Center, Department of Obstetrics and Gynecology, Perelman School of Medicine, University of Pennsylvania, Philadelphia, Pennsylvania, United States of America; 21 Department of Experimental Therapeutics, University of Texas MD Anderson Cancer Center, Houston, Texas, United States of America; 22 Department of Medicine, Indiana University Melvin and Bren Simon Comprehensive Cancer Center, Indiana University School of Medicine Indianapolis, Indiana, United States of America; 23 Eppley Institute for Cancer Research, Fred & Pamela Buffett Cancer Center, University of Nebraska Medical Center, Omaha, Nebraska, United States of America; Brigham and Women's Hospital, UNITED STATES

## Abstract

Metastasis is responsible for 90% of human cancer mortality, yet it remains a challenge to model human cancer metastasis *in vivo*. Here we describe mouse models of high-grade serous ovarian cancer, also known as high-grade serous carcinoma (HGSC), the most common and deadliest human ovarian cancer type. Mice genetically engineered to harbor *Dicer1* and *Pten* inactivation and mutant p53 robustly replicate the peritoneal metastases of human HGSC with complete penetrance. Arising from the fallopian tube, tumors spread to the ovary and metastasize throughout the pelvic and peritoneal cavities, invariably inducing hemorrhagic ascites. Widespread and abundant peritoneal metastases ultimately cause mouse deaths (100%). Besides the phenotypic and histopathological similarities, mouse HGSCs also display marked chromosomal instability, impaired DNA repair, and chemosensitivity. Faithfully recapitulating the clinical metastases as well as molecular and genomic features of human HGSC, this murine model will be valuable for elucidating the mechanisms underlying the development and progression of metastatic ovarian cancer and also for evaluating potential therapies.

## Author summary

Rarely does an experimental model fully replicate the clinical metastases of a human malignancy. Faithfully representing the clinical metastases of human high-grade serous ovarian cancer with complete penetrance, coupled with histopathological, molecular, and genomic similarities, these mouse models, particularly one harboring mutant p53, will be vital to elucidating the underlying pathogenesis of human ovarian cancer. In-depth understanding of the development and progression of ovarian cancer is crucial to medical advances in the early detection, effective treatment, and prevention of ovarian cancer. Also, these robust mouse models, as well as cell lines established from the mouse primary and metastatic tumors, will serve as useful preclinical tools to evaluate therapeutic target genes and new therapies in ovarian cancer.

## Introduction

Ovarian cancer is diagnosed predominantly at an advanced stage with widespread peritoneal metastases, resulting in a poor prognosis and high mortality [1–3]. Among the ovarian cancer types, high-grade serous ovarian cancer, also known as high-grade serous carcinoma (HGSC), is the most prevalent histotype of ovarian cancer, constituting more than 60% of all epithelial ovarian cancers [4–6]. Moreover, HGSC accounts for more than 80% of advanced-stage ovarian cancers and over 70% of all ovarian cancer deaths [6, 7]. Thus, HGSC is the most common and deadliest ovarian cancer [8].

Metastasis is a characteristic clinical feature of human HGSC [9]. While a small minority (~20%) of HGSC cases are diagnosed in early stages (stage I and II), a large majority (~80%) of HGSC cases are diagnosed in advanced stages (stage III and IV) [6–8], when tumors have already metastasized throughout the pelvic and peritoneal cavities, including the omentum and peritoneal membrane [9]. Advanced metastatic nature of HGSC leads to a relapse of cancer and poor overall survival [10, 11]. Therefore, a better understanding of the mechanisms underlying the metastasis of HGSC is critical to more effective treatment against advanced-stage HGSC. Metastatic HGSCs have been modeled in mice [7, 12]. Several mouse models, harboring gene mutations linked to ovarian cancer, produce HGSCs originating in the ovary or fallopian tube with moderate rates of penetrance [12]. However, despite the development of putative precursor lesions and primary tumors, peritoneal metastases are generally scarce or absent in these models [7].

In a previous study, we generated a double-knockout (DKO) mouse model of HGSC by inactivation of *Dicer1* and *Pten* [13]. This mouse model develops HGSC arising from the fallopian tube and exhibits peritoneal metastases. While this model resembles human HGSC in many aspects, it does not harbor p53 mutations. Mutations in the p53 gene are the singular molecular feature of the cancer genome in HGSC, observed in 96% of HGSC cases [14–16]. The p53 gene (*TP53* for humans; *Trp53* for mice) encodes a protein that functions as a tumor suppressor, which arrests cell-cycle progression, induces apoptosis, and triggers senescence [17, 18]. It is also one of the most frequently mutated genes in human cancer [19], as 50% of human malignancies carry p53 mutations [18], evidently suggesting that mutations in the p53 gene can cause cancer. Given the established role of p53 as a tumor suppressor and its common mutations in cancer, it is generally viewed that p53 mutations are likely an early genetic event driving HGSC development [15, 20]. Certainly, *TP53* mutations can occur early in tumorigenesis, including ovarian cancer. High levels of p53 proteins, indicative of p53 mutations, are frequently observed in serous tubal intraepithelial carcinoma (STIC), a putative precursor lesion in the fallopian tube [21–23].

Despite this obvious implication of the importance of p53 mutations in ovarian cancer, it is still unclear whether p53 mutations initiate and enable HGSC development. Genetic studies indicate that p53 mutations alone may not give rise to HGSC development. Li-Fraumeni syndrome is an inherited tumor-susceptibility disorder caused by germline p53 mutations [24, 25]. Li-Fraumeni patients are prone to developing a diverse array of tumors, including breast tumors, brain tumors, adrenocortical carcinomas, and soft tissue tumors [26]. Rarely, however, do these patients develop ovarian cancer [26]. Similarly, germline *TP53* mutations are rare in ovarian cancer patients [27]. Likewise, HGSC development is rare in mouse models with inactivation of the p53 gene or harboring mutant p53 alone [7, 28–30].

To model more closely human HGSC and elucidate the role of p53 mutation in ovarian cancer, we have incorporated mutant p53 into DKO mouse model, generating triple-mutant (TKO) mice [31]. Here we describe the comprehensive phenotypic, histopathologic, and molecular characterizations of DKO and TKO mouse models, which robustly and reliably represent the clinical metastases and genomic features of human HGSC.

## Results

### A mouse model with mutant p53 develops peritoneal metastatic HGSC with 100% penetrance

Given the vital role of p53 in tumor suppression and its frequent mutations in human HGSC, it was suggested that p53 mutations play a crucial role in the development, progression, or both [20]. To examine the significance of p53 mutations in HGSC, mutant p53 was integrated to generate a mouse model of HGSC genetically more closely associated with human HGSC. In this model, mice harboring a p53 mutant (*p53* ^R172H^) was bred to *Dicer1-Pten* double-knockout (DKO) mice (*Dicer1* ^flox/flox^ *Pten* ^flox/flox^ *Amhr2* ^cre/+^), a mouse model forming HGSC [13]. This breeding adds a conditionally activating p53 mutation to DKO mice, generating triple-mutant (TKO) mice: *p53* ^LSL-R172H/+^ *Dicer1* ^flox/flox^ *Pten* ^flox/flox^ *Amhr2* ^cre/+^. The murine p53^R172H^ is equivalent to human p53^R175H^, one of the most common p53 mutations in HGSC [16].

**Fig 1:**
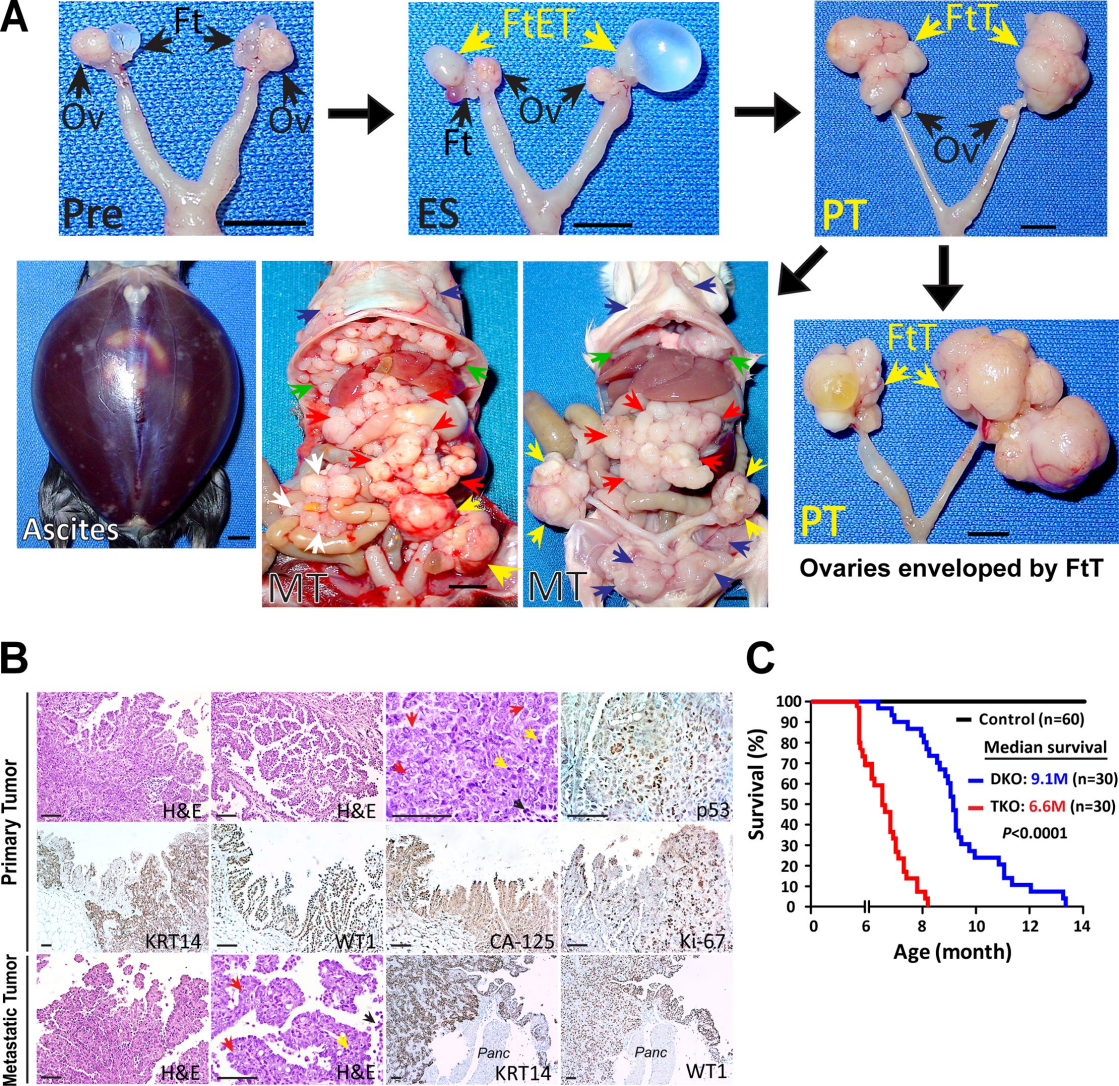
A mouse model with mutant p53 develops metastatic HGSC. **A. A stepwise progression of metastatic HGSCs in TKO mice.** At a premalignant stage (Pre), the fallopian tubes (Ft) form small cysts, which give rise to early tumors (FtET) at early stage (ES). The early-stage fallopian tube tumors (FtET) grow to become large fallopian tube tumors (FtT), while the ovaries (Ov) remain intact. The primary fallopian tube tumors (PT; FtT) (yellow arrows) expand and spread, enveloping the ovaries and disseminating throughout the pelvic and abdominal cavities to the omentum (red arrows), peritoneum (blue arrows), mesentery (white arrows), and diaphragm (green arrows). All mice develop widespread metastatic tumors (MT) with severe hemorrhagic ascites, leading to death. Scale bar, 0.5 cm. **B. Histopathologic characterization of primary and metastatic HGSCs from TKO mice.** The tumors display characteristic histopathological features of HGSC: a papillary, slit-like structure, along with a solid growth pattern, in which cancer cells exhibit intense mitosis (yellow arrows), abundant apoptosis (black arrows), pleomorphic and enlarged nuclei (red arrows), and prominent nucleoli with irregular chromatin aggregates (red arrows). In addition, mouse tumors are positive for several HGSC markers, including p53, WT1 (Wilms tumor 1), CA-125 (MUC16), and Ki-67 (MIB-1), and also are distinctively positive for the epithelial marker KRT14 (cytokeratin 14). Primary Tumor: primary fallopian tube tumors; Metastatic Tumor: omentum metastatic tumors; Panc: pancreas. H&E, hematoxylin & eosin. Scale bar, 100 μm. **C. Survival curves of TKO and DKO mice.** Survival of TKO mice is significantly shorter than that of DKO mice. The median survival is 6.6 months for TKO mice (n = 30) and 9.1 months for DKO mice (n = 30) (HR [hazard ratio] = 4.3; 95% confidence interval [CI], 2.3–8.2; Log rank test, *P*<0.0001). Control, 30 *Dicer1* ^flox/flox^ *Pten* ^flox/flox^ *Amhr2* ^*+*/+^ mice for DKO mice and 30 *p53* ^LSL-R172H/+^ *Dicer1* ^flox/flox^ *Pten* ^flox/flox^ *Amhr2* ^+/+^ mice for TKO mice.

All TKO mice progressively develop HGSC with 100% metastatic penetrance, as observed similarly in DKO mice [13] (Fig 1). According to the examinations of 263 TKO mice, tumors begin to form in the fallopian tube between 1–2 months after birth (Fig 1A; S1A Fig). The fallopian tube tumors steadily grow to envelop the ovaries and simultaneously spread throughout the pelvic and abdominal cavities (Fig 1A). Adjacent to the reproductive organs, the pelvic surface is covered with metastatic tumors. Inside the abdominal cavity, the tumors disseminate along and across the peritoneum. Most notably, HGSC metastasizes to the omentum, a fat-rich apron of peritoneal tissue that covers the intestines and abdominal cavity, one of the most common sites of metastasis in women with ovarian cancer [9]. The peritoneal metastases are widespread and abundant, extensively covering the surface of the peritoneal cavity, including the diaphragm and mesentery. These pervasive peritoneal metastases invariably lead to ascites and ultimately cause deaths (100% mortality).

Though extensive and ubiquitous, the metastases in these mice are confined to pelvic and abdominal cavities (stage III), a common metastatic pattern in women with HGSC [9]. Also, these metastatic tumors rarely invade the deeper layers (parenchyma) of organs (bowels, livers, and stomach) inside the peritoneal cavity, as observed similarly in ovarian cancer patients. In women with advanced ovarian cancer, the confinement of tumors inside the peritoneal cavity and minimal penetration into the abdominal organs allow a surgical resection of primary and metastatic tumors as part of treatment [9]. Tumors can metastasize outside the peritoneal cavity, spreading to the lungs (stage IV) in mice (S1B Fig), indicative of a hematogenous dissemination of ovarian cancer [32]. Lung metastasis, however, is rarely observed in TKO mice. Typically, mice die of widespread peritoneal metastases prior to lung metastasis. Additionally, highly vascularized tumors, aggressive and widespread peritoneal metastases, and consistent formation of hemorrhagic ascites in these mice also corroborate the prominent role of angiogenesis in human ovarian cancer [33]. Collectively, the overall features of pelvic and peritoneal metastases in mice strikingly represent the clinical characteristics of human HGSC [9].

Histologically, these primary and metastatic tumors are characteristic of HGSC (Fig 1B). The tumors display a papillary, slit-like structure in which cancer cells exhibit frequent mitosis, abundant apoptosis, pleomorphic and enlarged nuclei, and prominent nucleolus—the characteristic histopathological features of HGSC. These tumors are also positive for known markers for ovarian cancer, such as Wilms tumor 1 (WT1), p53, Ki-67 (MIB-1), and CA-125 (MUC16), and are positive for the epithelial marker cytokeratin 14 (KRT14) in both primary and metastatic tumor tissues (Fig 1B; S1A Fig).

### Addition of mutant p53 markedly shortens survival in mice

Overall, both TKO and DKO mice followed a similar course of tumor development and progression: formation of primary tumors (HGSC) in the fallopian tube, subsequent spread to the ovary and metastases throughout the peritoneal cavity, development of ascites accompanying peritoneal metastases, and resulting death [13] (Fig 1A). The tumor burdens at death were also comparable between both models. Despite the indistinguishable tumor phenotypes, however, the striking difference was mouse survival. TKO mice died markedly earlier than DKO mice. The median survival was 6.6 months of age (5.1–8.2) for TKO mice (n = 30) and 9.1 (6.3–13.3) for DKO mice (n = 30) (p<0.001) (Fig 1C). This significant decrease in survival suggests that mutant p53 may accelerate tumor development and progression.

### Genetic relevance of mutant p53, *Pten*, and *Dicer1* to human HGSC

**Fig 2:**
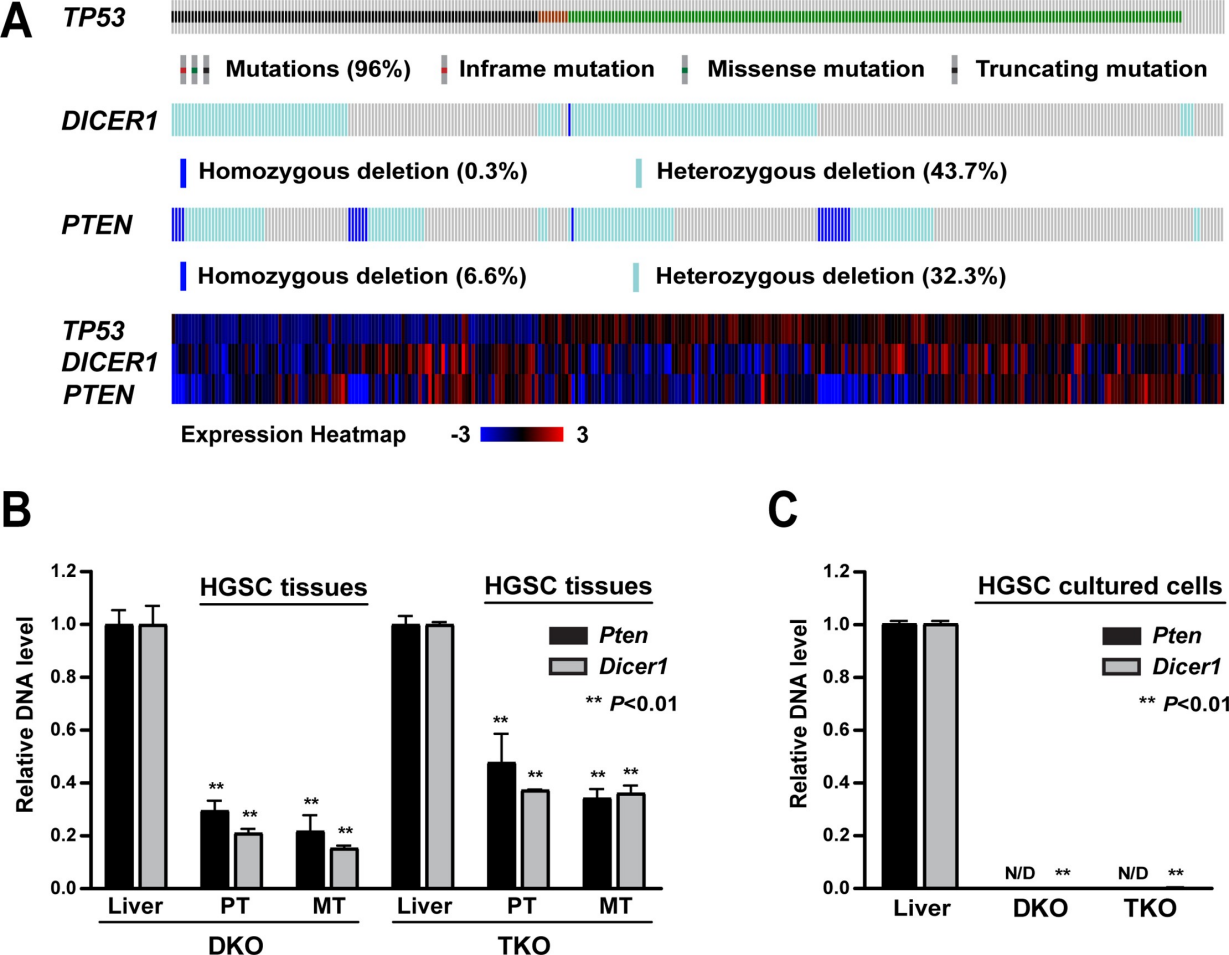
Genetic relevance of mutant p53, *Pten*, and *Dicer1* in human HGSC. **A.** *TP53* mutation as well as *DICER1* and *PTEN* homozygous and heterozygous deletion frequencies in 316 human HGSCs (TCGA, 2011) [14] analyzed in cBioportal [85]. Each column represents an individual patient sample. *TP53* is mutated in 96% of HGSC cases, while *DICER1 and PTEN* hemizygous deletions are detected in 43.7% and 32.3%, respectively. Expression heatmap depicts altered mRNA expression levels of *TP53*, *DICER1*, and *PTEN* in corresponding samples. **B-C.** Quantitative PCR showing the extent of *Pten* and *Dicer1* deletion in (B) primary (PT) and metastatic (MT) HGSC tissues from DKO and TKO mice and (C) cell lines established from DKO and TKO primary HGSC tissues. Two individual HGSC tissues for PT and MT and two HGSC cell lines are used for each model. The DNA levels of *Pten* and *Dicer1* in HGSC tissues and cell lines are measured as fold changes relative to the DNA levels of these genes in liver tissues in DKO and TKO mice. N/D, not detectable.

The genetic link of mutant p53 to human ovarian cancer is highlighted in the finding that 96% of HGSC cases harbor p53 mutations [14]. *PTEN* deletion is observed in 38.9% of HGSC cases (homozygous loss: 6.6%; heterozygous loss: 32.3%) (Fig 2A). In addition, PI3K signaling is activated in 45% of HGSC cases [14]. As PTEN is a major inhibitor of PI3K signaling, loss of *Pten* leads to activation of PI3K signaling [13], supporting the relevance of *Pten* loss in modeling human HGSC.

DICER1 has also been linked to human ovarian cancer. Low expression of DICER1 is associated with advanced stages and reduced survival in human ovarian cancer [34], suggesting that DICER1 may act as a potential tumor suppressor. Yet the functional role of DICER1 in ovarian cancer remains unclear. In human HGSC, homozygous deletion of *DICER1* (*DICER1* ^-/-^) is extremely rare (0.3%: 1/316 patients) (Fig 2A). However, nearly half of HGSC cases (43.7%) exhibit a single-copy loss of *DICER1* (*DICER1* ^+/-^). Intriguingly, it is also suggested that, though copy-number analysis may not be sensitive enough to discern, human HGSC tissue may harbor a small number of *DICER1*-null cells (*DICER1* ^-/-^) along with the vast majority of *DICER1*-positive cells [35].

Similarly, DKO and TKO HGSC tissues appeared to contain *Dicer1*-positive cells (Fig 2B). Presence of *Dicer1*-positive cells is likely attributable to the fact that tumor tissues comprise not only cancer cells but also non-cancer cells, such as endothelial cells, immune cells, and cancer-associated fibroblasts [36]. Accordingly, when DKO and TKO HGSC tissues were cultured to establish cell lines, these cultured HGSC cells were homozygous for *Dicer1* deletion (*Dicer1* ^-/-^), indicating that HGSC cells lack *Dicer1* (Fig 2C). Thus, mouse HGSC tissues appear to harbor a mixture of *Dicer1*-null cells and *Dicer1*-positive cells, as postulated in human HGSC tissue [35]. Collectively, these findings suggest that *DICER1* deletion may play a role in the development of human HGSC.

A gene-expression heatmap also indicates decreased mRNA levels of *DICER1 and PTEN* in corresponding patient samples with homozygous and heterozygous deletions of these genes (Fig 2A). Interestingly, around 20% (63/316 patients) of HGSC cases show co-occurrence for *PTEN* and *DICER1* loss (Fig 2A), implying relevance of these genes to human HGSC.

### Marked chromosomal abnormalities and genomic instability in mouse and human HGSCs

**Fig 3:**
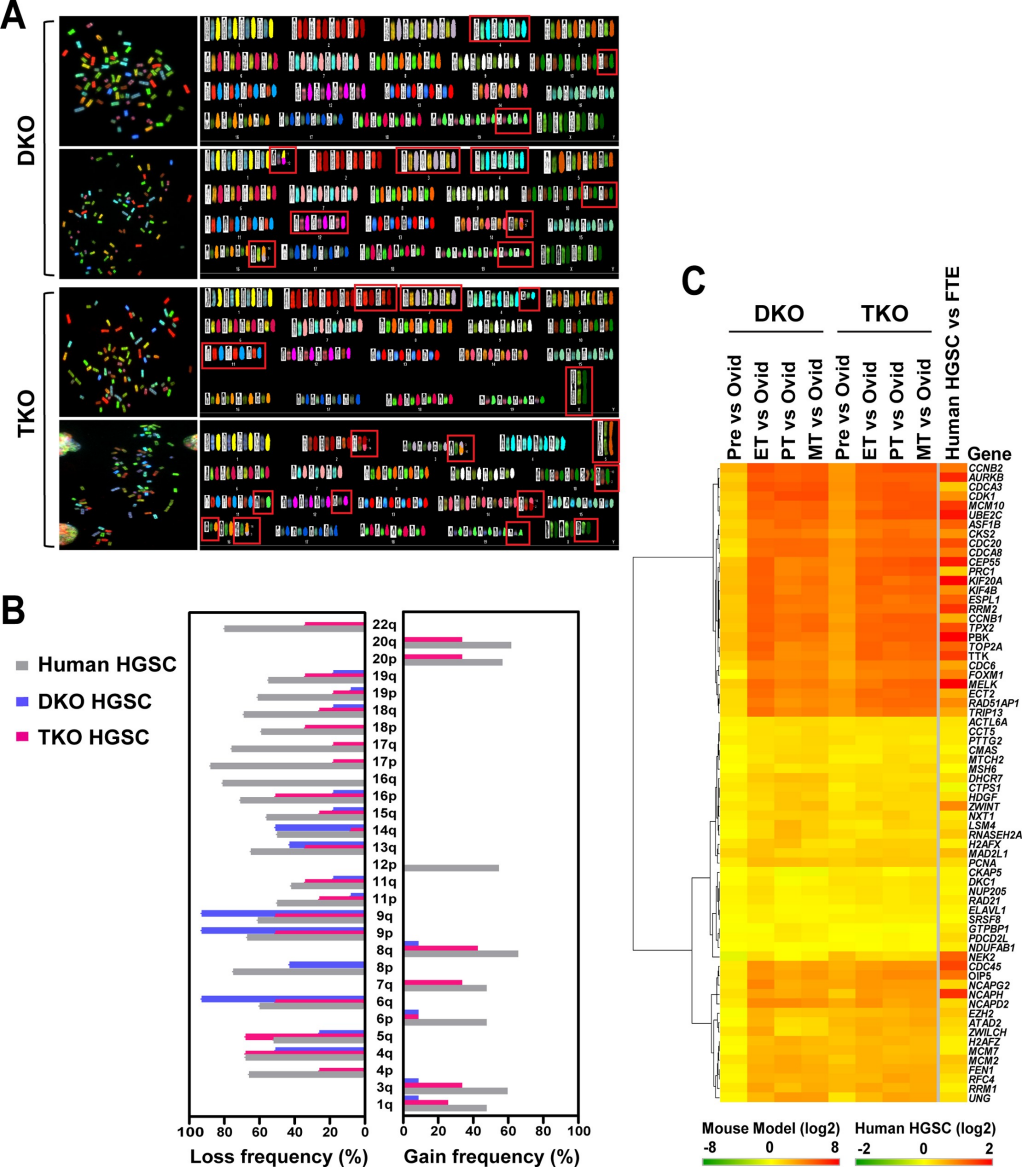
Marked chromosomal abnormalities and genomic instability in mouse and human HGSCs. **A. Karyotype analysis of DKO and TKO cell lines by spectral karyotyping (SKY).** Both DKO and TKO HGSC cells display abnormal near-tetraploid and aneuploid karyotypes. Numerical (chromosome gain/loss) and structural (translocation and deletion) alterations are marked in red boxes. Each chromosome is displayed as duplicated chromosomes at metaphase. Overall, TKO cells are represented by higher levels of karyotypic heterogeneity (differences among metaphase spreads within the same cell line), recurrent translocations, and unique chromosomal aberrations than DKO cells. **B. Comparison of chromosome gains and losses between mouse and human HGSCs.** There are substantial overlaps in chromosome-arm gains and losses between mouse and human HGSCs. Both DKO and TKO HGSC cells exhibit comparable patterns of chromosome-arm loss frequencies in comparison with human HGSC [14], whereas there is a higher concordance in gain frequencies between mouse TKO HGSC and human HGSC, indicative of greater genomic instability in TKO cells. **C. CIN70 gene expression analysis of chromosomal (genomic) instability.** A heatmap compares mRNA expression of the CIN70 gene signature, a gene set whose net overexpression correlates with chromosomal instability [37], between mouse DKO, TKO, and human HGSCs. Chromosomal instability is comparable between human HGSC, early-stage (ET), and advanced-stage (PT and MT) mouse HGSCs. There are statistically significant positive correlations in CIN70 signature expression between human HGSC and all stages of mouse HGSCs (Pre, ET, PT, and MT) (Spearman’s rank correlation coefficient: 0.69–0.73; *P*<0.0001). Ovid: normal mouse fallopian tubes; Pre: premalignant-stage mouse fallopian tubes; ET: early-stage mouse fallopian tube HGSC; PT: primary mouse (fallopian tube) HGSC; MT: metastatic mouse HGSC; and FTE: (human) fallopian tube epithelium. CIN70: 70 genes associated with chromosome instability (CIN). HGSC, high-grade serous carcinoma (high-grade serous ovarian cancer).

The genomic hallmark of human HGSC is a prominent degree of genomic instability [14, 20]. To assess the genomic similarity between human and mouse HGSCs, chromosomal abnormalities were examined in TKO and DKO cell lines, which were derived from primary HGSCs formed in these mouse models. Spectral karyotyping (SKY) chromosome analysis on each representative line of DKO and TKO cell lines revealed: (i) numerical chromosomal alterations, which include aneuploidy (gain or loss of individual chromosomes) and near-tetraploidy (4n; four sets of chromosomes); and (ii) structural abnormalities such as translocations, duplications, and deletions (Fig 3A). Overall, these chromosomal alterations were more pronounced in TKO cells than in DKO cells. Also, many of these chromosomal alterations correspond to chromosomal gains or losses observed in human HGSCs (Fig 3B; S1 Table).

In addition, chromosomal instability (CIN) was examined in murine (TKO and DKO) HGSC tissues and further compared to human HGSC, by analyzing the expression of 70 genes (CIN70) associated with aneuploidy [37]. As illustrated in the heatmap (Fig 3C), the overexpression of the CIN70 gene signature was evident in early-stage and advanced-stage murine HGSCs as well as in human HGSC, indicative of widespread chromosomal abnormalities. Also, there were positive correlations of CIN70 signatures between murine and human HGSCs (Spearman’s rank correlation coefficient: 0.69–0.73; *P*<0.001). These chromosomal aberrations extrapolated by CIN70 overexpression are consistent with the high levels of aneuploidy observed in TKO and DKO HGSC cell lines.

**Fig 4:**
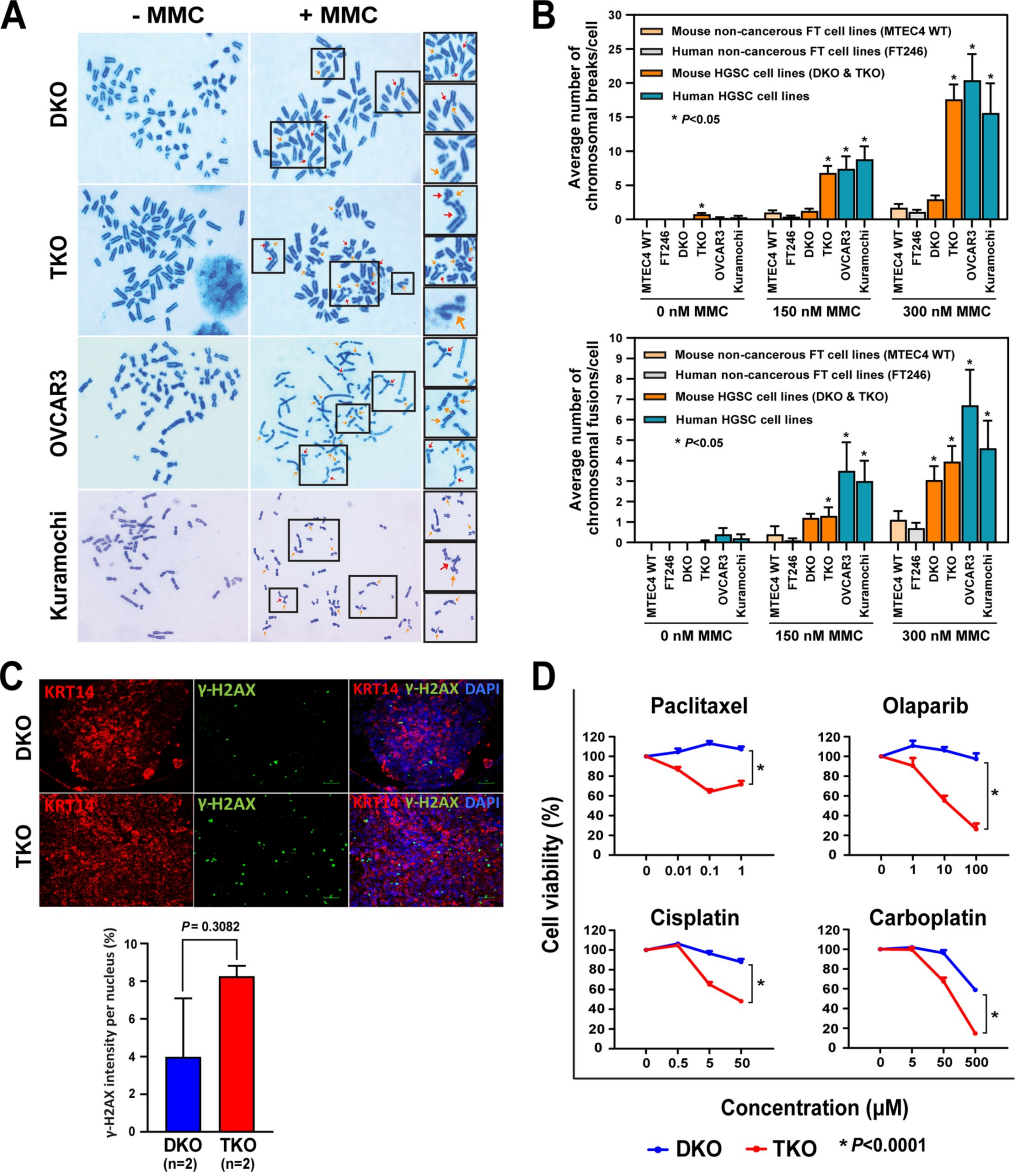
Impairment of DNA repair in mouse DKO, TKO, and human HGSCs. **A-B. Mitomycin C (MMC) assay assessing DNA repair ability. A.** Widespread chromosomal breaks (orange arrows) and fusions (red arrows) in mouse DKO and TKO HGSC cell lines as well as human HGSC cell lines, following treatment with mitomycin C (MMC). **B.** Number of breaks and fusions per cell in mouse (DKO and TKO) and human (OVCAR3 and Kuramochi) HGSC cell lines exposed to 0, 150, and 300 nM MMC, compared with control mouse (MTEC4) and human (FT246) non-cancerous fallopian tube cells. * Indicates significant *P* values (*P*<0.05) in comparisons between mouse or human non-cancerous and cancer cell lines. MTEC4, immortalized mouse tubal epithelial cells. FT246, immortalized human fallopian tube secretory epithelial cells (FTSEC). **C. DNA double-strand breaks in DKO and TKO HGSC tissues.** Immunofluorescent staining of γ-H2AX (green), a marker for DNA double-strand breaks, and KRT14 (red), a marker for mouse HGSC, was performed on formalin-fixed paraffin-embedded tissue sections generated from early-stage DKO and TKO HGSCs. Two HGSC tissues each for DKO and TKO mice were examined. **D. Chemosensitivity of DKO and TKO cell lines.** Cell viability measured by MMT assay following treatment with a chemotherapy drug (paclitaxel, cisplatin, carboplatin, or olaparib) reveals chemosensitivity of DKO and TKO cells to all drugs with markedly higher sensitivity in TKO cells (*P*<0.0001).

Marked chromosomal aberrations, characteristic of genomic instability, are linked to defective DNA repair [20, 36, 38, 39]. Thus, to assess the DNA-repair capability in mouse HGSCs, a chromosomal breakage assay was performed using mitomycin C (MMC), a DNA-damaging agent [40, 41]. Various concentrations of MMC (0, 150, and 300 nM) were treated to mouse HGSC (DKO and TKO) and human HGSC (OVCAR3 and Kuramochi) cell lines—as well as non-cancerous mouse (MTEC4) and human (FT246) cell lines derived from normal mouse and human fallopian tubes, respectively [42–44]. Increasing concentrations of MMC stimulated a greater number of chromosomal abnormalities, such as chromosomal breaks and fusions, in both mouse and human HGSC cell lines (Fig 4A), suggesting markedly impaired DNA-repair ability. In contrast, genome integrity was preserved in non-cancerous mouse and human cell lines (Fig 4B).

Besides chromosomal aberrations, DNA damage was examined by immunofluorescent staining of γ-H2AX, a marker for double-strand DNA breaks, in early-stage DKO and TKO HGSC tissues. Overall, γ-H2AX expression was prevalent in both DKO and TKO HGSCs (Fig 4C). Additionally, though variable, TKO HGSC tissue tends to exhibit higher levels of γ-H2AX staining than DKO tissue, suggesting a greater degree of impaired DNA repair and genomic instability in TKO HGSC. Overall, mouse HGSCs exhibit widespread genomic instability resembling that of human HGSC.

### Chemosensitivity of mouse HGSCs

We next examined chemosensitivity in mouse HGSC cells. TKO and DKO cell lines were treated with varying concentrations of chemotherapy drugs commonly used in ovarian cancer treatment, including carboplatin, cisplatin, paclitaxel, and olaparib, a poly (ADP-ribose) polymerase (PARP) inhibitor. Cell viability measured by MTT (3-(4,5-dimethylthiazol-2-yl)-2,5-diphenyltetrazolium bromide) assay indicated that both TKO and DKO cells were sensitive to chemotherapy drugs at certain concentrations (Fig 4D). Moreover, TKO cells were significantly more sensitive to these drugs than DKO cells (*P*<0.0001). Unlike DKO cells, TKO cells consistently harbored a sub-G1 population, indicative of apoptosis [45] (S2 Fig). Presence of a sub-G1 population may be attributable to a higher level of genomic instability in TKO cells, which may also give rise to a higher sensitivity to chemotherapy drugs. Curiously, despite the lack of *Brca1* mutations, TKO cells were acutely sensitive to the PARP inhibitor olaparib. This acute sensitivity to olaparib may be attributable to high genomic instability (Fig 3 and Fig 4A–4C) as well as marked dysregulation of homologous recombination (HR) and DNA repair signaling observed in TKO HGSCs.

### *In vivo* transplantability of mouse HGSCs

To evaluate whether mouse HGSCs can be reproduced *in vivo*, primary tumors, metastatic tumors, and ascites from TKO mice, as well as HGSC cell lines derived from primary tumors, were inoculated intraperitoneally (*i*.*p*.) into immunocompetent control mice: mixed C57BL/129Sv mice or DKO control littermates (*Dicer1* ^flox/flox^ *Pten* ^flox/flox^ *Amhr2* ^+/+^), both of a genetic background similar to that of TKO mice. With a varying degree of incidence (0–100%) and time (1.6–3.2 months) for tumor formation, mice injected with TKO HGSC cells developed widespread peritoneal HGSCs with hemorrhagic ascites, a phenotype and histopathology confirmed to be identical to that of TKO mice (S1C Fig; S2 Table), as observed similarly in DKO HGSCs [13]. The *in vivo* reproducibility of HGSC reinforces the strength of this mouse HGSC robustly modeling metastatic human HGSC.

In addition, this *in vivo* tumorigenic potential of mouse HGSC cells presents an encouraging possibility that murine metastatic HGSC could also be successfully modeled in commonly used strains of immunocompetent mice, such as C57BL/6 (Black 6). However, the variable *in vivo* tumor-forming ability of TKO HGSC tissue cells and TKO HGSC cultured cells (0–100%) reflect variation in the transplantability of TKO HGSCs. Thus, for development of a useful syngeneic cell line model for HGSC, it would be important to select a TKO cell line that can reliably produce HGSC when inoculated in commonly used strains of immunocompetent mice. This syngeneic model of HGSC will be immensely useful for genetic and pharmacologic studies investigating potential targets and therapies in ovarian cancer.

### Mutant p53 hastens tumor onset and enhances metastasis during the course of HGSC

**Fig 5:**
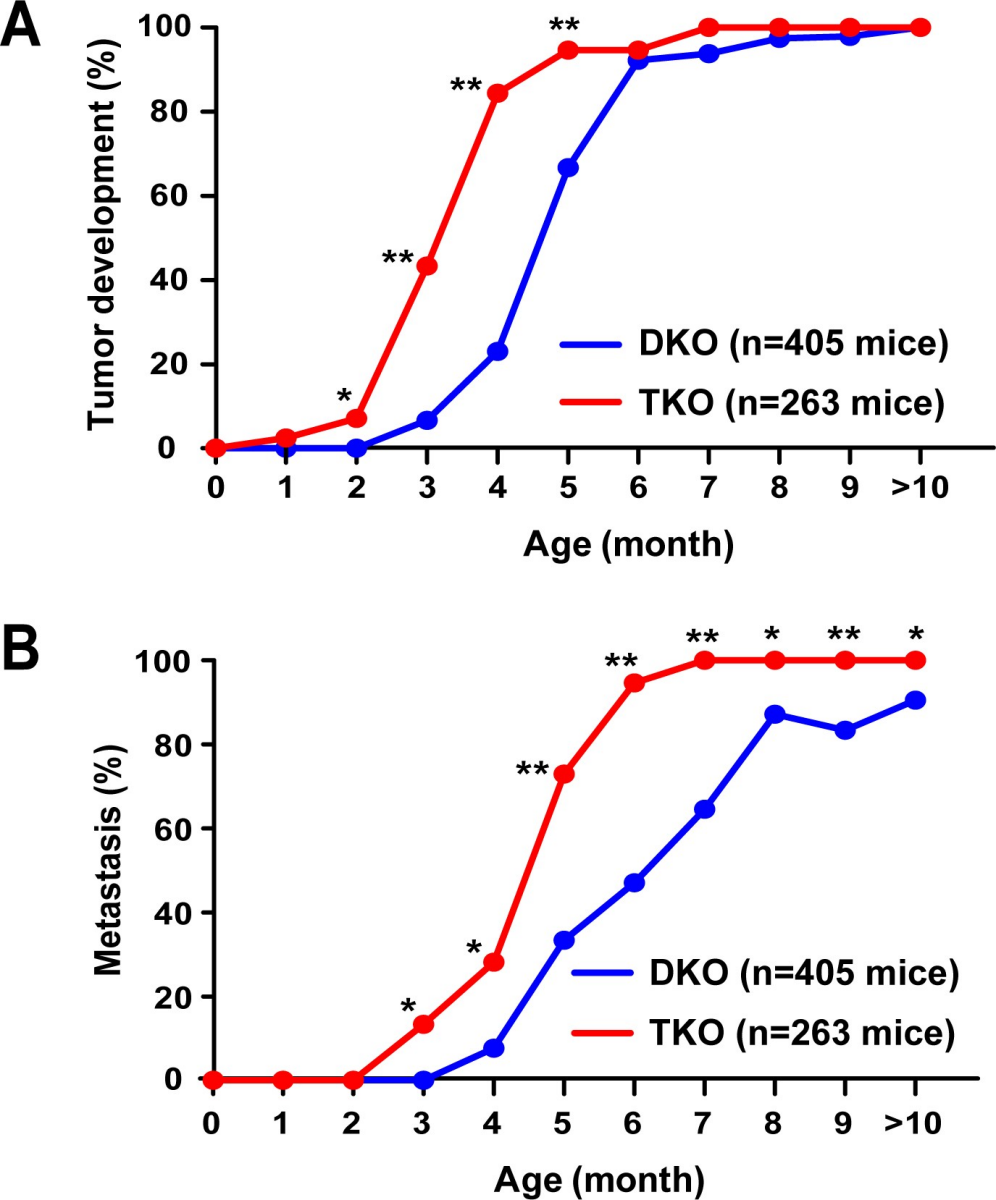
Early tumor onset and rapid metastatic dissemination in TKO mice. **A. Tumor incidence.** Incidence of primary fallopian tube tumor formation in DKO (n = 405) and TKO (n = 263) mice. TKO mice begin to form tumors as early as 2 months of age, and the incidence increases rapidly and significantly at 3–5 months of age, relative to DKO mice. **B. Incidence of metastasis.** The proportion of TKO mice bearing metastatic tumors is significantly higher than that of DKO at 3–4 months of age with increasing differences at 5–7 months of age. * *P*<0.01, **P<0.0001.

To elucidate the impact of mutant p53 on tumor progression, we first examined the ages at which primary fallopian tube tumors would emerge in TKO and DKO mice. The incidence of primary and metastatic tumors was profiled during the course of HGSC development and progression in TKO and DKO mice. A total of 263 TKO mice and 405 DKO mice were examined from 0 to >10 months of age to monitor tumor development and metastasis (Fig 5; S3 Table). As plotted (Fig 5A), primary tumors emerged earlier in TKO mice than in DKO mice. In TKO mice, early-stage fallopian tube tumors began to appear at as early as 2 months of age (7.1%: 3/42 mice). By 3 months after birth, fallopian tube tumors were present in close to half of TKO mice (43.3%: 13/30 mice), by 4 months, 84.4% (27/32), and by 5 months, nearly all of TKO mice (94.6%: 35/37). In contrast, the earliest age at which fallopian tube tumors appeared in DKO mice was 3 months after birth (6.6%: 1/15 mice), with gradual increases at 4 months (23.1%: 3/13 mice) and at 5 months (66.7%: 20/30). These findings suggest that an earlier occurrence of HGSC likely contributes to the shortened survival of TKO mice (Fig 1C).

Besides the earlier tumor incidence, metastasis was also accelerated in TKO mice. As primary tumors emerged earlier, metastasis would be expected to occur earlier in TKO mice than in DKO mice. There was, however, more than a proportional increase of early metastatic incidences expected from the earlier tumor occurrences in TKO mice (Fig 5B; S3 Table). In TKO mice, a rise in metastatic incidence had sharply escalated, beginning at 3–4 months of age and peaked at 5–7 months (*P*<0.0001). This rapid rise in the incidence of metastasis suggests that not only does mutant p53 expedite tumor onset, but it also facilitates metastasis. Mutant p53 (R172H) is known to make tumors more metastatic in mouse models [29, 46], and it is associated with a poor prognosis in cancer patients [47]. Therefore, mutant p53 would account for rapid tumor progression and metastasis, which ultimately led to a worse prognosis in TKO mice. Together, a marked acceleration of TKO death would be attributable to an early onset in tumor development as well as enhanced metastasis, both driven by mutant p53.

### Cell proliferation is augmented in mouse HGSC cells harboring mutant p53

A prominent tumor suppressive function of p53 is inhibition of cell proliferation through arresting the cell cycle [18]. A missense mutation (e.g., R172H) in the p53 gene is known to result in a loss of its tumor-suppressive function, in a dominant negative manner, by inhibiting the function of the remaining wild-type (WT) p53 allele [48]. Thus, mutant p53 could induce a loss of WT p53 function, driving cell proliferation.

**Fig 6:**
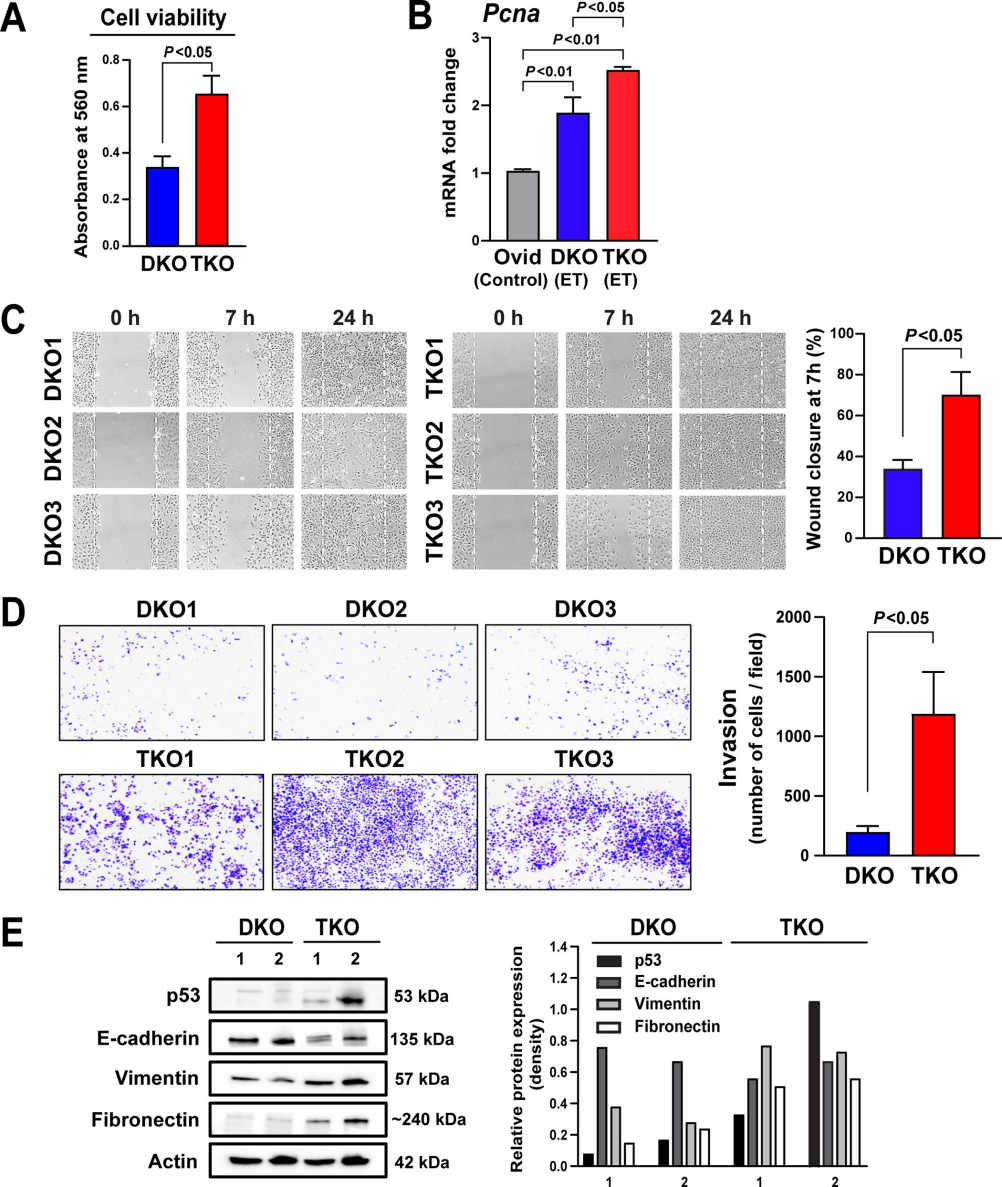
Enhanced proliferation and invasiveness in TKO tumors. **A. Cell viability.** MTT assay indicates that cell lines derived from TKO HGSC have higher viability and proliferative potential than those derived from DKO HGSC. **B. Cell proliferation.** Real-time PCR measures mRNA expression levels of *Pcna* in early-stage tumors (ET) from DKO and TKO mice. Both models show high *Pcna* levels with a greater increase in TKO tumors. **C. Cell migration.** Migration ability of DKO and TKO cells is examined using a wound healing assay. Representative pictures show cell migration at 0 hours, 7 hours, and 24 hours. A higher cell migration rate is evident in TKO cells, compared with DKO cells, at 7 hours after scratch. **D. Invasion.** Invasion assay reveals that TKO cells exhibit markedly higher capacity to invade through Matrigel than DKO cells. Invaded cells were stained with crystal violet and counted as number of cells per field. **E. EMT marker expression.** Western blot shows a decrease in E-cadherin and increases in vimentin and fibronectin protein expression in TKO cells, relative to DKO cells. In DKO cells, p53 protein is undetectable, as WT p53 protein would be rapidly degraded by MDM2-mediated proteasome degradation. In contrast, TKO cells exhibit an enhanced expression of p53 protein, as mutant p53 protein is less sensitive to degradation and accumulates in the cell. Two DKO and two TKO cell lines were used in this analysis. Band densities were measured by ImageJ and expressed relative to Actin. EMT: epithelial-to-mesenchymal transition.

To determine whether mutant p53 promotes cell proliferation, cell viability was compared between DKO and TKO HGSC cell lines. MTT assay showed that a significantly higher number of viable cells were present in TKO cells than in DKO cells (Fig 6A). In human HGSC, mutant p53 is thought to activate the FOXM1 network, promoting cell-cycle progression and thus cell proliferation [14]. Accordingly, proliferation-related FOXM1 target genes, including *Foxm1*, *Plk1*, and *Aurkb*, as well as the cell-proliferation marker *Pcna* were overexpressed in mouse HGSCs with more robust expression in TKO mice (Fig 6B).

Also notable in TKO cells was rapid cell-cycle progression. Flow cytometry analysis indicates that TKO cells progress more rapidly to G1 phase than DKO cells (S2 Fig). After thymidine-nocodazole block, TKO cells exhibited a significantly larger cell population in G1 and a significantly smaller cell population in G2/M than DKO cells (e.g., 2 h: G1, 40.2% in TKO vs 24.4% in DKO; G2/M, 36.1% in TKO vs 49.6% in DKO; *P*<0.05), suggesting faster progression of cell cycles in TKO cells. Collectively, these findings suggest that TKO cells harboring mutant p53 are more proliferative than DKO cells with no mutations in the p53 gene. This enhanced cell proliferation may have also contributed to an earlier development of HGSC in TKO mice.

### TKO cells harboring mutant p53 exhibit enhanced invasion and migration

Apart from inducing the loss of WT p53 function, mutant p53 can also acquire oncogenic function, enhancing tumor aggressiveness and metastatic potential [19, 49]. Thus, the accelerated metastasis in TKO mice may also be attributable to mutant p53. To evaluate the metastatic potential of mutant p53, we compared the ability of TKO and DKO cells for invasion and migration. Wound healing assay showed that TKO cells more rapidly migrated across to fill the cell-free area in the plate well than DKO cells (wound closure at 7h after scratch was 70% in TKO vs 34% in DKO; *P*<0.05) (Fig 6C), suggesting enhanced migratory capacity. In the invasion assay, TKO cells showed significantly higher ability to penetrate the Matrigel-coated chamber, as indicated by intense and abundant crystal violet staining, compared with DKO cells (*P*<0.05) (Fig 6D), suggesting increased invasive ability.

In addition, epithelial-to-mesenchymal transition (EMT), a process involved in metastasis, appeared to be augmented in TKO cells relative to DKO cells, as indicated by altered expression of EMT-related genes, including E-cadherin, vimentin, and fibronectin (Fig 6E). Together, these findings suggest that besides promoting cell proliferation, mutant p53 may also enable tumor aggressiveness and metastatic potential.

### Dysregulation of critical signaling pathways associated with HGSC

**Fig 7:**
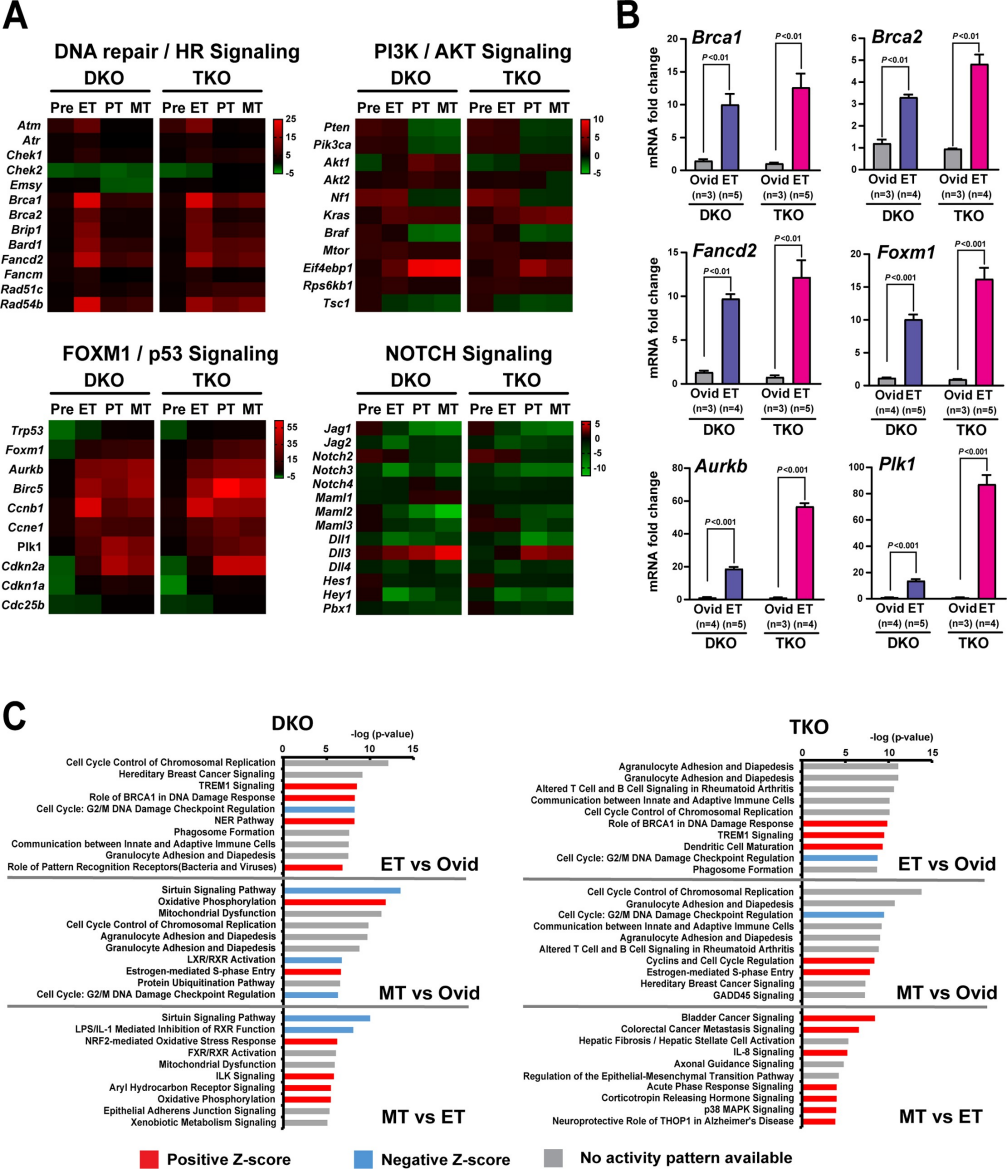
Dysregulation of critical signaling pathways associated with HGSC. **A. Commonly altered signaling pathways in HGSC.** Heatmap analysis of gene expression from RNA sequencing data from DKO and TKO tissues at different stages of HGSC development. Mouse HGSCs (ET, PT, and MT) exhibit markedly altered gene expression levels for the signaling pathways known to be dysregulated in human HGSC [14]. **B.** Real-time PCR validation of representative genes using randomly selected early-stage HGSCs (ET) from DKO and TKO mice. Markedly increased mRNA levels of genes involved in homologous recombination (HR) and FOXM1 signaling in DKO and TKO tumors with higher expression in TKO tumors. **C. Potential pathways of HGSC development and metastasis.** Ingenuity Pathway Analysis (IPA) of differentially expressed genes from DKO and TKO tumors reveals signaling pathways dysregulated in early-stage HGSC and metastatic HGSC. TOP10 altered pathways are presented per each comparison and ranked by the negative log of the p value of the enrichment score. The color scheme is based on Z scores, with activation in red, inhibition in blue, and undetermined directionality in gray. Ovid (oviduct): normal mouse fallopian tubes; Pre: premalignant-stage mouse fallopian tubes; ET: early-stage mouse fallopian tube HGSC; PT: primary mouse (fallopian tube) HGSC; MT: metastatic mouse HGSC.

The comprehensive gene-mutation profiling analysis of a large collection of human HGSC cases reveals, to our surprise, that HGSC patients harbor few recurrent potential driver mutations, except mutations in p53 (*TP53*: 96%) and *BRCA1/2* (22%) [14]. Instead of common individual mutations, several key oncogenic signaling pathways are commonly altered in these HGSC cases: RB and PI3K signaling, NOTCH signaling, homologous recombination (HR) alterations, and FOXM1 signaling. This finding reinforces the view that cancer is driven by altered core pathways as much as individual driver mutations [50, 51]. Accordingly, these core pathways in human HGSC were also widely altered in mouse HGSCs, as indicated by altered expression levels of genes associated with these pathways (Fig 7A & 7B). Notably, there was a profound dysregulation of genes involved in DNA repair/HR signaling (Fig 7A & 7B), in line with widespread genomic instability observed in mouse HGSCs. Additionally, the components of the FOXM1 network, including *Foxm1*, *Plk1*, and *Aurkb*, were overexpressed in mouse HGSCs and more robustly in TKO mice (Fig 7A & 7B). Interestingly, *Brca1* and *2* expression was elevated in both mouse HGSCs with higher levels of expression in TKO HGSC (Fig 7A & 7B). A high level of *BRCA1* expression, likely indicative of profound DNA damage, was also observed in sporadic HGSCs and was associated with a poor prognosis [52, 53]. Also, gene-set enrichment analysis (GSEA) revealed significant correlations for the genes upregulated as well as downregulated between mouse and human HGSCs (S3 Fig). Collectively, these findings suggest that inactivation of *Dicer1* and *Pten*, accompanied by mutant p53, activates critical pathways faithfully giving rise to HGSC.

### Diverse pathways associated with HGSC development and metastasis

Metastasis is primarily responsible for ovarian cancer mortality [9]. One salient feature of these mouse models is a remarkable resemblance to the clinical metastases of human HGSC. Thus, this unique feature may offer insights into the potential pathways crucial to metastasis in human HGSC. Pathway analysis, however, has revealed that several pathways altered in metastatic tumors (MT)—including cell cycle control of chromosomal replication and G2/M DNA damage checkpoint regulation—are also dysregulated in early-stage tumors (ET), when each tumor type compared with normal fallopian tubes using gene-expression profiles from RNA sequencing (Fig 7C). A similar pattern in pathway alterations between MT and ET suggests an interesting possibility that HGSC, albeit at an early stage, may have already acquired a vital property enabling metastasis. Curiously, MT-specific pathways, gleaned from the analysis of gene-expression profiles between MT and ET, were vastly different between DKO and TKO metastatic tumors. These diverse molecular alterations, giving rise to the same phenotype (i.e. the same cancer type), may reflect the genetic and molecular heterogeneity observed among individual patients with advanced ovarian cancer [14]. Distinct pathways also point to diverse mechanisms enabling and driving metastasis in HGSC, which would pose an immense challenge facing the treatment of advanced ovarian cancer.

## Discussion

### Robust mouse modeling of ovarian cancer metastasis

Metastasis is primarily responsible for most cancer deaths [54, 55]. Ovarian cancer, particularly high-grade serous ovarian cancer (HGSC), is commonly diagnosed in advanced stages with widespread peritoneal metastases, leading to a poor prognosis [1, 2, 7]. Crucial to devising the effective treatment of advanced cancer would be a lucid understanding of the mechanisms driving metastasis. As human cancer is often diagnosed at an advanced stage, however, the natural history of metastatic tumors—how a tumor initiates, grows, and progresses to metastatic cancer—remains poorly understood for most metastatic human malignancies, including ovarian cancer. One way to overcome this limitation is to create animal models that replicate the metastases of human malignancies. Disappointingly, generation of such cancer mouse models mimicking human cancer metastasis has been challenging [56]. When gene mutations are engineered into mice, a tumor or a putative precursor lesion may form in a primary tissue of interest, but metastasis is often lacking [7, 12].

In the current study, addressing the challenges of modeling cancer metastasis, we present comprehensive phenotypic and molecular characterizations of mouse models—TKO and DKO mice—for metastatic human HGSC. It is striking that mutations of three (*p53*, *Dicer1*, and *Pten*) or two (*Dicer1* and *Pten*) genes reliably give rise to peritoneal metastases, which are 100% penetrant in mice and closely resemble the clinical metastases of human HGSC. Besides phenotypic and histopathological similarities to human HGSC, these mouse HGSCs also exhibit a marked degree of chromosomal abnormality and genomic instability—the genomic hallmarks of human HGSC.

Faithfully representing human HGSC, these models will serve as useful preclinical tools for evaluating potential therapies and also offering vital insights into early detection and prevention of ovarian cancer. For instance, metabolomic alterations are one of the defining features of malignancies, including ovarian cancer [57, 58]. Harnessing the stepwise tumor development and progression in TKO model, our recent study has identified a panel of 29 serum metabolites that can distinguish early-stage HGSC from normal controls and also from advanced-stage HGSC with high accuracy, thus offering useful guidance on early detection of ovarian cancer [59].

### Crucial signaling pathways causing metastatic HGSC

One surprising revelation of the TCGA genomic studies on human malignancies is vast heterogeneity of gene mutations among individual patients for the same cancer type, indicated by low rates of recurrent mutations within each cancer type [60, 61]. For instance, in human HGSC, aside from p53 and *BRCA* mutations, few mutations are common to HGSC cases [14]. Individual HGSC cases exhibit mutation profiles vastly different from one another [14]. Instead, ovarian cancer is characterized with frequent copy number alterations, indicative of a chromosomally unstable malignancy [62–64]. Undoubtedly, cancer is a genetic disease driven by gene mutations [50]. The overall lack of common driver mutations, however, also suggest that cancer is a pathway disease [65]. That is, discrete sets of gene mutations could activate common pathways leading to the same malignancy [51, 60]. Fitting this view, human HGSCs exhibit dysregulation of several potentially critical common pathways [14]: RB and PI3K signaling, NOTCH signaling, homologous recombination (HR), and FOXM1 signaling. Similarly, these signaling pathways are markedly altered in HGSCs from DKO and TKO models (Fig 7), supporting the molecular similarity between these mouse HGSCs and human HGSC.

Individual genes—*p53*, *Dicer1*, and *Pten*—in these mouse models are also shown to be relevant to human HGSC. With mutant p53 (*TP53*) found in nearly all cases of human HGSC (96%), homozygous and heterozygous deletions of *DICER1* (44.0%) and *PTEN* (38.9%) are also common genetic events in human HGSC (Fig 2A) [14]. However, despite the individual relevance of these genes to human HGSC, generally, human HGSC may not be a direct consequence of mutations in these genes. Rather, these mouse models highlight crucial signaling pathways driving metastatic HGSC: that is, loss of *Dicer1* and *Pten* with mutant p53 alters critical pathways leading to the development and progression of metastatic HGSC.

### Cancer-promoting role of mutant p53 in HGSC

These mouse models also provide insights into the role of mutant p53 in HGSC. Mutations in the p53 gene (*TP53*), particularly mutant p53, are found in nearly all cases of HGSC [14, 15], which naturally leads to a notion that p53 mutations would give rise to HGSC development. However, genetic mutations of the p53 gene alone do not appear to be sufficient to cause HGSC, as evidenced by the lack of ovarian cancer in patients with Li-Fraumeni syndrome harboring germline p53 mutations and the scant development of HGSC in mouse models with p53 mutations [7, 24, 26, 27, 29]. Also, a mouse model lacking p53 mutations is fully capable of developing metastatic HGSC resembling human HGSC [13].

Yet our current study supports that dysregulation of p53 signaling is critical to the development and progression of HGSC. The augmented proliferation driven by mutant p53 may facilitate an early onset of HGSC in TKO mice (Fig 6A & 6B; Fig 5A). Mutant p53 has been suggested to promote human HGSC by activating the FOXM1 network, which may lead to cell-cycle progression and proliferation as well as impaired DNA repair [14]. The components of the FOXM1 network, including *Foxm1*, were all overexpressed in mouse tumors and more pronouncedly in TKO HGSCs (Fig 7A & 7B). Mutant p53 and FOXM1 have also been linked to precipitating genomic instability [37, 66, 67]. Therefore, the enhanced genomic instability evident with mutant p53 in TKO model may also contribute to the early onset of HGSC in TKO mice. In addition to facilitating tumor development, mutant p53 is likely vital to tumor progression in HGSC. Mutant p53 significantly augmented aggressiveness and metastatic potential of HGSC in TKO mice (Fig 6C–6E), accelerating tumor progression (Fig 5B). Resulting from the early development and rapid progression of HGSC driven by mutant p53, TKO mice die nearly three months earlier than DKO mice (Fig 1C). Collectively, our findings suggest that the cooperation between p53 mutations and other genetic and molecular alterations may be necessary to enable HGSC development followed by rapid progression and metastasis of HGSC.

### Fallopian tube origin of metastatic HGSC in mouse models

Undoubtedly, the fallopian tube is the origin of metastatic HGSCs in TKO and DKO mice. In the absence of the fallopian tubes, the ovaries alone fail to form tumors; the fallopian tubes are capable of developing tumors in the absence of the ovaries [13]. Surprisingly, however, TKO and DKO HGSCs, albeit epithelial cancers, do not appear to arise in fallopian tube epithelium—rather seem to form in fallopian tube stroma (S1A Fig) [13]. In fact, this would not be surprising, as the *Amhr2*-Cre (*Amhr2* ^*cre/+*^) is active in the stroma of the reproductive tract, including the fallopian tube, uterus, and cervix [68]. Curiously, a lineage-tracing study of *Amhr2* in the mouse uterus shows that, while most of *Amhr2*-lineage cells differentiate into stromal cells in the uterus, a fraction of these *Amhr2*-Cre-expressing cells, likely stromal-lineage stem cells, become epithelial cells in the uterus after parturition [69]. Thus, it is plausible that inactivation of *Dicer1* and *Pten* in stem cells in the fallopian tube stroma could result in the formation of HGSC, a malignant epithelial cancer equipped with full metastatic potential.

This notion, however, is in contrast with the current prevailing view on the cell of origin of human HGSC. In early 2000s, insights based on microscopic observations of the fallopian tubes and ovaries prophylactically removed from high-risk women carrying *BRCA1/2* mutations had led to a hypothesis [70, 71]: that a non-invasive microscopic tumor lesion formed in the fallopian tube epithelium, termed serous tubal intraepithelial carcinoma (STIC), may be a cell of origin of human HGSC [21, 72]. These STIC lesions coexist with HGSC lesions in patients diagnosed with HGSC [73, 74]. A lineage analysis of the gene mutations between STIC and HGSC concurrently observed in patients also suggests that STIC may have evolved into HGSC [75]. Though the STIC origin of human HGSC has been widely embraced [76, 77], the causal relationship between STIC and metastatic peritoneal HGSC remains to be corroborated with more concrete evidence [7, 78]. As HGSC is diagnosed predominantly at an advanced stage, HGSC is inextricably linked to metastasis. Therefore, if STIC is a bona fide precursor lesion of metastatic HGSC, it will need to be rigorously established that STIC lesions can reliably transform into HGSC and, more crucially, metastasize to become peritoneal HGSCs [7].

In summary, robustly modeling the clinical metastases of human HGSC with genomic and molecular similarities, TKO and DKO mice can offer unique opportunities to elucidate the pathogenic mechanisms underlying metastatic HGSC, including tumor initiation, development, progression, and metastatic dissemination. These mouse models can be valuable for discovering novel factors (endogenous or exogenous) vital to the development and progression of metastatic human HGSC.

## Materials & methods

### Ethics statement

Mouse use and experiments involving mice were performed in accordance with the guidelines approved by the Institutional Animal Care and Use Committee (IACUC) at Indiana University School of Medicine (approval number: 18093).

### Generation of triple-mutant (TKO) mice and double-knockout (DKO) mice

Triple-mutant (TKO) mice (*p53* ^LSL-R172H/+^ *Dicer1* ^flox/flox^ *Pten* ^flox/flox^ *Amhr2* ^cre/+^) and double-knockout (DKO) mice (*Dicer1* ^flox/flox^ *Pten* ^flox/flox^ *Amhr2* ^cre/+^) were generated as described previously [13, 31].

### Mouse and human cell lines

Primary mouse ovarian cancer cell lines were established from primary (fallopian tube) HGSCs developed in DKO and TKO mice, referred to as DKO and TKO cell lines, respectively. Briefly, primary tumor tissues from DKO and TKO mice were washed with PBS, physically dissociated by mincing with a blade, cultured, and passaged until cell lines were established. DKO and TKO cells were cultured in Dulbecco’s Modified Eagle’s Medium (DMEM)/F12 supplemented with 10% fetal bovine serum (FBS) and 1% antibiotic-antimycotic (AA) solution at 37°C with 5% CO_2_. DKO (p53 WT) and TKO (p53 R172H) cell lines used in the experiments were derived from primary tumor tissues from 3 individual mice per each mouse model, and designated as DKO1, DKO2, DKO3 and TKO1, TKO2, TKO3, respectively. MTEC4 (immortalized mouse tubal epithelial cells) and FT246 (immortalized human fallopian tube secretory epithelial cells [FTSEC]) were provided by Drs. Joanna E. Burdette and Ronny Drapkin, respectively. FT246 cells were immortalized by stable expression of human telomerase reverse transcriptase (hTERT), CDK4^R24C^, and p53 shRNA, which silences p53 expression [44]. The MTEC4 cell line (WT p53) was established by culture of oviductal tissue from d16 CD1 mice and spontaneous immortalization from continuous passages [79]. The MTEC4 and FT246 cells were cultured as described previously [44, 79]. In OVCAR3 cell line, the p53 gene harbors a G to A point mutation at nucleotide 743, resulting in a change of Arg (R) (CGA) to Gln (Q) (CAG) at codon 248 (p53 R248Q/R248Q); in Kuramochi cell line, the p53 gene has a G to T mutation at nucleotide 841, leading to an Asp (D) (GAC) to Tyr (Y) (TAC) change at codon 281 (p53 D281Y) [80].

### MTT assay

Cell viability was analyzed using the MTT (3-(4, 5-dimethylthiazol-2-yl)-2, 5-diphenyltetrazolium bromide) assay. DKO and TKO cells were seeded in 96-well cell culture plates in 6 replicates at 5x10^3^ cells/well. After the cells were allowed to grow for 2 days, MTT solution (5 mg/ml in sterile PBS) was added to each well followed by incubation at 37°C for 4 hours. Subsequently, 200 μl of DMSO was added to resuspend formazan (MTT metabolic product). Cell viability was measured using a SpectraMax Plus plate reader at 560 nm absorbance and adjusted to reference absorbance at 670 nm. Also, cell viability was examined after treatment of cells with various concentrations of chemotherapy drugs. Olaparib and cisplatin were purchased from Fisher Scientific (Waltham, MA, USA). Carboplatin and paclitaxel were purchased from Sigma-Aldrich (St. Louis, MO, USA).

### Wound healing assay

Wound healing assay was employed to analyze the migration ability of DKO and TKO cells. DKO and TKO cell lines were seeded in 6-well culture plates at 1x10^5^ cells/well. When the cells reached 100% confluency, a scratch was made with a sterile blue tip to mimic a wound. After washing with PBS, the cells were added with fresh medium. Migration of the cells to close the scratch zone was observed at 0, 7, and 24 hours after the scratch. The data were recorded as the length of a cell-free area and expressed as a difference in % of wound closure at 7 hours.

### Invasion assay

Matrigel invasion assay was used to assess the invasion ability of DKO and TKO cells. Using Biocoat Matrigel invasion chambers (Corning, NY 14831 USA), DKO and TKO cells were seeded at 1x10^5^ cells/well in the upper chamber containing serum-free media. The lower chamber was filled with the culture medium containing 10% FBS. The cells were incubated for 16 hours at 37°C with 5% CO2. Cells that remained in the upper chamber were removed after incubation. The bottom chamber surface was fixed with 4% formaldehyde solution and stained with 0.1% gentian violet (RICCA, Batesville, IN, USA) for 15 minutes. The inserts were air-dried and repeated in triplicate. The invaded cells were counted per field of view from 2 random fields and statistically analyzed.

### Western Blot analysis

DKO and TKO cells were grown to confluency and then lysed in RIPA buffer containing protease and phosphatase inhibitors (Thermo Fisher Scientific, Waltham, MA, USA). Protein content was determined using Bradford assay (Sigma). Protein extracts were mixed with 2x Laemmli sample buffer and heated to 95°C for 5 min. Samples were subjected to SDS–PAGE using 4–12% (vol/vol) gradient NuPage gels and subsequently transferred to a nitrocellulose membrane. The membrane was then blocked with TBST-5% skim milk for 1 hour at room temperature (RT) prior to incubation with a primary antibody in TBST-5% BSA (1:500 or 1:1,000 dilution) overnight at 4°C. Next day, the membrane was washed extensively with TBST and incubated with the appropriate peroxidase conjugated secondary antibody in TBST-5% BSA for 1 hour at RT. Proteins were visualized by enhanced chemiluminescence (ECL) using Clarity western ECL substrate (Bio-Rad, Hercules, California, USA). The following primary antibodies were used: anti-p53 antibody (rabbit; sc-6243; Santa Cruz Biotechnology, Dallas, TX, USA), anti-E-cadherin (rabbit; #4065; Cell Signaling Technology, Danvers, MA, USA), anti-vimentin (rabbit; #5741; Cell Signaling Technology), anti-Fibronectin antibody (mouse; F6140; Sigma-Aldrich), and anti-β-actin (mouse; C-4, sc-47778, Santa Cruz Biotechnology).

### Immunohistochemistry and immunofluorescence

Primary and metastatic tumor tissues from DKO and TKO mice were fixed in 10% (vol/vol) formalin at RT for 24 hours, embedded in paraffin, sectioned at 5 μm, and mounted on slides (Superfrost Plus, Fisher Scientific). Hematoxylin and eosin (H&E) staining was performed on paraffin sections according to standard protocols. Immunohistochemistry was performed as described previously [13]. The following antibodies were used for immunohistochemical and immunofluorescent analyses: anti-p53 antibody (rabbit; sc-6243; Santa Cruz Biotechnology); anti-KRT14 antibody (rabbit; #PRB-155P; BioLegend, Dedham, MA, USA); anti-Wilms Tumor Protein (WT1) antibody (rabbit; ab89901; Abcam, Cambridge, MA, USA); anti-Ki-67 antibody (mouse; #550609; BD Biosciences, San Jose, CA, USA); anti-phospho-Histone H2AX (Ser139) antibody (clone JBW301; mouse; Millipore Sigma, Kankakee, IL, USA); and anti-CA125 (MUC16) antibody (rabbit; a gift from Dr. Robert Bast Jr.)

### Cell synchronization and FACS analysis

Cells were arrested in mitosis with a thymidine-nocodazole block, and cell synchrony was monitored by flow cytometry of propidium iodide-stained cells. Cell cycle synchronization was adapted from the protocol of Whitfield et al [81]. Briefly, cells were blocked for 24 hours with 2 mM thymidine (Fisher Scientific), released for 3 hours by washing out the thymidine and adding fresh DMEM/F12 medium with 1% FBS and 1% AA. Then the cells were blocked with 100 ng/ml nocodazole (Sigma-Aldrich) for 12 hours to arrest all cells in mitosis. The cells were released from nocodazole block into G1 synchronously by washing out nocodazole and adding fresh medium. The cells were harvested at 0, 2, 4, and 8 hours after release, washed with PBS, and fixed overnight in 70% ethanol. Fixed cells were then centrifuged, washed, resuspended in PBS containing RNase A (100 μg/mL) and propidium iodide (50 μg/mL), and incubated for 20 minutes on ice and in the dark. Stained cells were analyzed using BD LSR II flow cytometer (BD Biosciences) and cell cycle population distribution was determined by ModFit LT software (Verity software house, Topsham, ME, USA).

### Quantitative PCR for genomic DNA

Genomic DNA was obtained from DKO and TKO HGSC tissues, cell lines, and liver tissue using Gentra Puregene Tissue Kit (Qiagen). Liver tissue does not express *Amhr2*-Cre and was thus used as a negative control. Purified genomic DNA was diluted to 100 ng/ul. Quantitative PCR was performed on specific primer sets using PowerUp SYBR Green Master mixture (Applied Biosystems) in QuantStudio 3 Real-Time PCR system (Applied Biosystems). The sequences of primers are described in S4 Table. DNA levels obtained with *Dicer1* negative and *Pten* negative primers were used as reference DNA levels (negative primers are located in upper intron/exon regions of floxed sites of *Dicer1* and *Pten*). Quantitative analysis for relative DNA levels was performed using the 2^-ΔΔCT^ method, which computes a fold change in DNA levels relative to control liver tissue.

### RNA preparation and sequencing

Total RNA was isolated at different stages of tumor development in DKO and TKO mice: fallopian tubes at premalignant stage (Pre), early-stage fallopian tube tumors (ET), primary fallopian tube tumors (PT), and metastatic tumors (MT). Additionally, RNA was isolated from normal fallopian tubes (Ovid, oviducts) of respective control mice for DKO and TKO mice. Total RNA was isolated using TRIzol (Invitrogen) and purified using RNeasy MinElute Cleanup Kit (Qiagen, Germantown, MD, USA), according to manufacturer’s instructions. Preliminary RNA quality and quantity were evaluated using a Nanodrop Spectrophotometer (ND-1000). RNA quality was further examined using a bioanalyzer (Agilent 2200 and 4200). Total RNA (1-1.2μg) was used for mRNA library preparation. Library preparation was performed using the TruSeq Stranded mRNA HT Library preparation kit (Illumina). The libraries were pooled at a concentration of 1nM and the sequencing analysis was performed using the NextSeq 75 (Illumina). Reads were aligned and gene counts were generated using STAR_2.5.3a [82]. For each library, read pairs uniquely aligned to the exon regions of each gene annotated on the genome were counted using featureCounts tool of subread package v.1.6.1 [83]. Normalized counts and differential expression analysis were conducted using DESeq2 v. 1.12.3 by providing these read counts as input. Genes with FDR < = 0.05 were considered significantly differentially expressed [84]. Fold changes were measured to quantify the expression levels of differentially expressed genes by comparing read counts of Pre, ET, PT, or MT relative to those of Ovid control.

### Quantitative RT-PCR

Approximately 2–3 μg of total RNA was used to synthesize cDNA with SuperScript IV Reverse Transcriptase (Invitrogen). Quantitative real-time PCR (qRT-PCR) was performed on cDNA with specific primer sets in PowerUp SYBR Green Master mixture (Applied Biosystems) using QuantStudio 3 system (Applied Biosystems). The sequences of primers are described in S4 Table. *Gapdh* and 36b4 (*Rplp0*) were used as the reference genes. Quantitative analysis for relative gene expression was performed using the 2^-ΔΔCT^ method, which computes a fold change in gene expression relative to control sample.

### Chromosomal instability (CIN) analysis

To analyze the chromosomal instability in mouse and human HGSCs using gene-expression profile [37], Euclidean Hierarchical Cluster analysis was performed on RNA-Seq RPKM log2 expression change values across CIN70 genes using a GENE-E software. CIN70 genes denote 70 genes whose expression is correlated with aneuploidy in cancer [37]. Analysis included CIN70 signatures from all stages of tumor development in DKO and TKO mice—premalignant stage fallopian tubes (Pre) as well as early-stage (ET), advanced primary (PT), and metastatic (MT) tumors—compared with normal fallopian tubes (Ovid) of control mice. Data from human HGSCs were obtained from TCGA [14] and were analyzed relative to normal human fallopian tubes. The Spearman rank correlation test was employed to analyze associations of CIN70 signatures between DKO, TKO, and human HGSCs.

### Spectral Karyotyping (SKY) analysis

For the SKY analysis, DKO and TKO cells were split a day before to get cells at log phase of growth. Next day, the cells were harvested by mitotic shake-off after colcemid treatment (0.1 μg/ml; 45 min) (Life Technologies, Carlsbad, CA, USA). Standard cytogenetic methods using 0.075 M potassium chloride and a methanol-acetic acid (3:1) fixative were employed. Metaphase spreads were prepared under optimized humidity conditions. Spectral karyotyping was performed using Mouse Spectral Karyotyping Kit (Applied Spectral Imaging, Migdal Haemek, Israel) according to the manufacturer’s protocol. Complete spectral karyotypes were defined for DKO and TKO cell lines (12 metaphases each). SKY analysis was carried out on single representative DKO and TKO lines selected, based on cell proliferation and migration ability, from 5 DKO and 5 TKO cell lines, which were derived from primary fallopian tube tumors from 5 DKO and 5 TKO mice, respectively.

### Chromosomal breakage analysis

Mouse (DKO and TKO) and human (OVCAR3 and Kuramochi) ovarian cancer cell lines as well as non-cancerous mouse (MTEC4) and human (FT246) fallopian tube epithelial cells were treated with mitomycin C (MMC) (Sigma-Aldrich), a DNA cross-linking agent, to induce chromosomal breakage, as described previously [41]. The cells were treated with 0, 50, and 150 nM of mitomycin C for 48 hours. After colcemid treatment, metaphase spreads were prepared and stained with Giemsa (Sigma-Aldrich). Chromosomal breaks and fusions were analyzed microscopically (x100 magnification).

### *In vivo* transplantation of mouse HGSCs

To examine the *in vivo* tumor-forming ability of HGSCs developed in TKO mice, cells were isolated from primary and metastatic HGSC tumors as well as ascites in TKO mice, and injected intraperitoneally into immunocompetent C57BL/129Sv mice. In addition, cell lines derived from primary HGSC tissues were also injected intraperitoneally into immunocompetent control mice lacking Cre expression (*p53* ^+/+^ *Dicer1* ^flox/flox^ *Pten* ^flox/flox^ *Amhr2* ^+/+^), whose genetic background is similar to that of TKO mice. Tumors developed from these injections were histologically examined after H&E staining.

### Statistical analysis

Statistical analyses were performed using a GraphPad Prism software (Version 8, GraphPad Software, Inc.). *P* values less than 0.05 were considered statistically significant. Data are shown as mean ± SEM from at least three independent experiments. One-way ANOVA, Chi-square (*x*^*2*^), and Student’s *t* test (two-tailed) were used to analyze differences between experimental groups. Log-rank test was employed to compare the natural lengths of survival between TKO and DKO mice.

## Supporting information

S1 Fig The cell of origin of HGSC and tumor property in TKO mice. **A. HGSC arises in fallopian tube stroma in TKO mice.** HGSC cells in the fallopian tube stroma exhibit distinct positive staining for the epithelial marker KRT14 (arrows), a marker for TKO HGSC. In contrast, epithelial cells in the fallopian tube show no evidence of tumor (arrowheads). **B. Lung metastases in TKO mice.** Lungs riddled with metastatic tumors in a TKO mouse that developed widespread peritoneal HGSCs. **C. *In vivo* recapitulation of TKO mouse HGSCs.** Tumors in the mesentery of immunocompetent mice, which developed from intraperitoneal injections of TKO HGSCs, exhibit the same histopathology characteristic of HGSCs observed in TKO mice (Fig 1B).
(TIF)

S2 Fig Comparison of cell cycles between DKO and TKO cells. A scheme (in a black box) shows synchronization of DKO and TKO cells by thymidine-nocodazole (Thy-Noc) block and subsequent cell cycle analysis using *fluorescence-activated cell sorting* (FACS). After synchronous release from Thy-Noc block, TKO cells progressed to G1 phase more rapidly than DKO cells, which seemed to have a delay in G2-to-M and M-to-G1 transitions compared with TKO cells (all *P* values <0.05). Interestingly, TKO cells also showed a sub-G1 population at all comparisons. Numbers inside the columns indicate cell population distribution (%) at each cell-cycle phase.
(TIF)

S3 Fig Gene Set Enrichment Analysis (GSEA) between mouse HGSCs and human HGSC. This analysis assesses similarity in gene expression between mouse (DKO and TKO) HGSCs and human HGSC from TCGA data [14]. Both DKO and TKO HGSCs (early-stage tumors, primary tumors, and metastatic tumors) were significantly enriched with the genes that are upregulated and downregulated in human HGSC. The representative enrichment plots indicate high significance of correlation between DKO ET and human HGSC (A) and between TKO ET and human HGSC (B) for the upregulated and downregulated genes. FDR<0.00001 (for all GSEAs between mouse HGSCs and human HGSC). ET, early-stage fallopian tube HGSC. NES, normalized enrichment score. FDR, false discovery rate.
(TIF)

S1 Table Chromosome gain and loss frequencies in human, mouse TKO, and DKO HGSCs. (DOCX)

S2 Table *In vivo* transplantation of mouse HGSCs. (DOCX)

S3 Table Tumor development and metastasis in DKO and TKO mice. (DOCX)

S4 Table Sequences of quantitative real-time PCR primers. (DOCX)

S5 Table Human data information for CIN70 analysis and GSEA. (DOCX)
