# Supplementary figures and images for: In vivo modeling of metastatic human high-grade serous ovarian cancer in mice
Source: PLoS Genet. 2020 Jun 4;16(6):e1008808. doi: 10.1371/journal.pgen.1008808 (PMC7297383; doi:10.1371/journal.pgen.1008808)

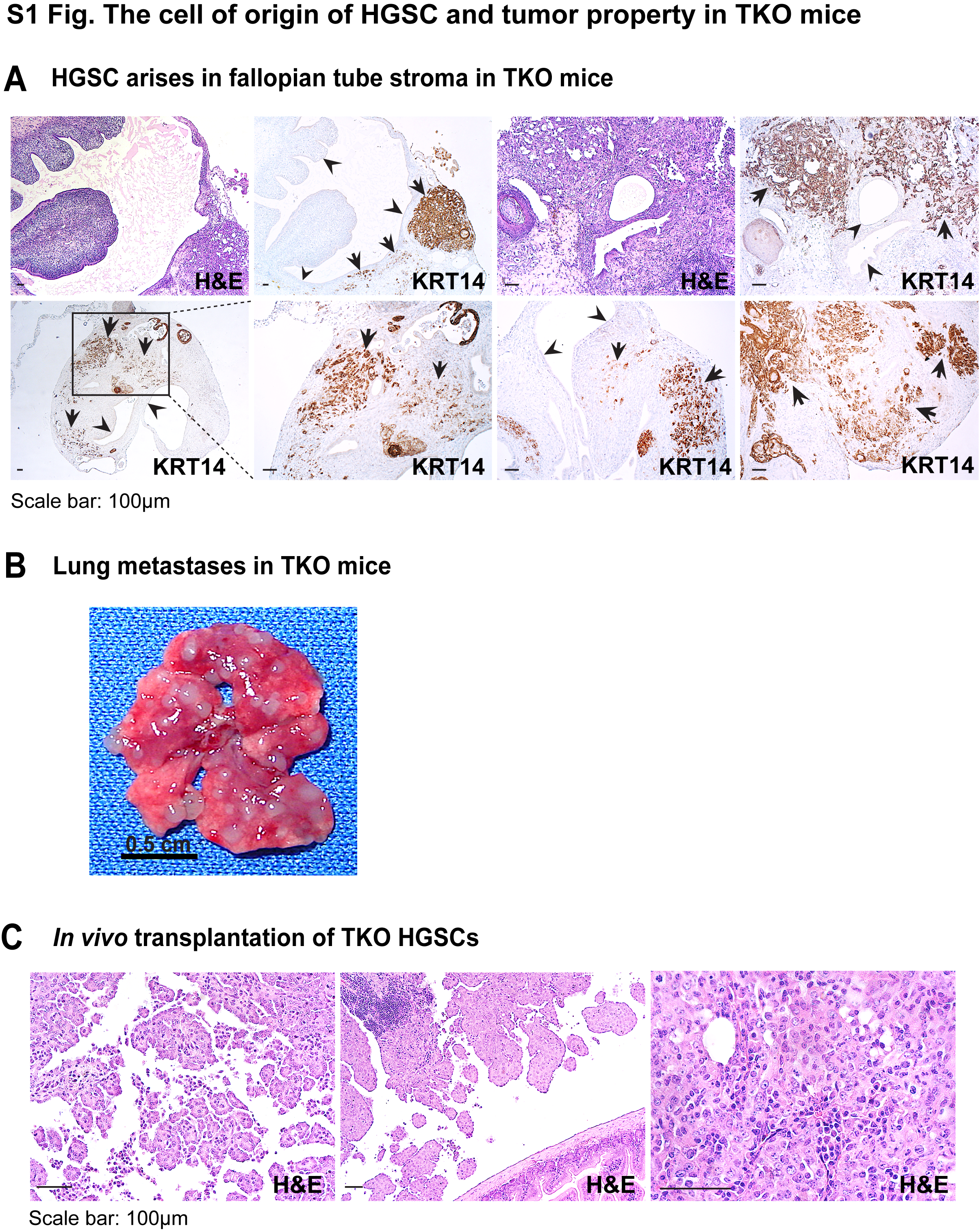

Supplement: S1 Fig — A. HGSC arises in fallopian tube stroma in TKO mice. HGSC cells in the fallopian tube stroma exhibit distinct positive staining for the epithelial marker KRT14 (arrows), a marker for TKO HGSC. In contrast, epithelial cells in the fallopian tube show no evidence of tumor (arrowheads). B. Lung metastases in TKO mice. Lungs riddled with metastatic tumors in a TKO mouse that developed widespread peritoneal HGSCs. C. In vivo recapitulation of TKO mouse HGSCs. Tumors in the mesentery of immunocompetent mice, which developed from intraperitoneal injections of TKO HGSCs, exhibit the same histopathology characteristic of HGSCs observed in TKO mice (Fig 1B). (TIF) [file pgen.1008808.s001.tif]

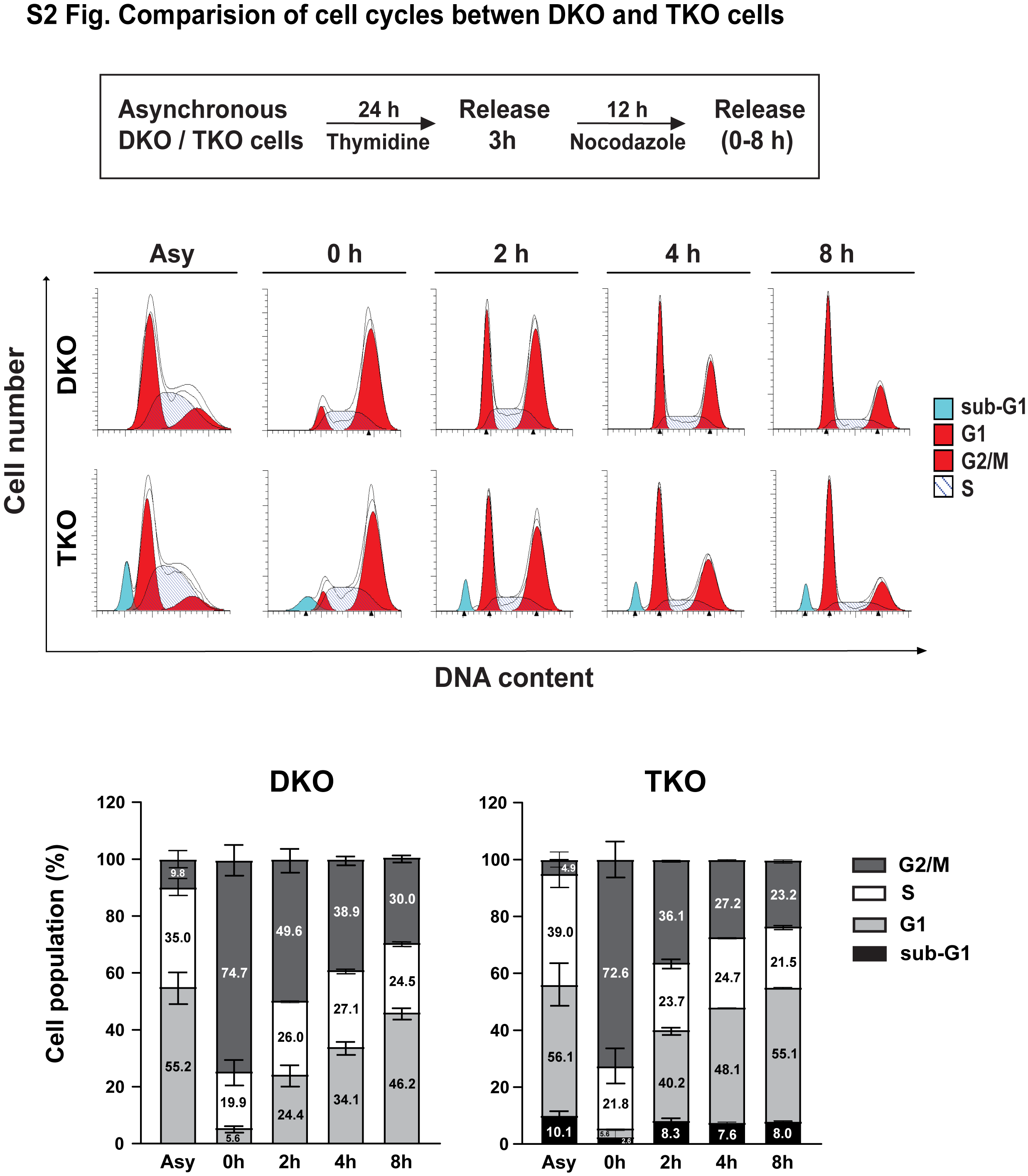

Supplement: S2 Fig — A scheme (in a black box) shows synchronization of DKO and TKO cells by thymidine-nocodazole (Thy-Noc) block and subsequent cell cycle analysis using fluorescence-activated cell sorting (FACS). After synchronous release from Thy-Noc block, TKO cells progressed to G1 phase more rapidly than DKO cells, which seemed to have a delay in G2-to-M and M-to-G1 transitions compared with TKO cells (all P values <0.05). Interestingly, TKO cells also showed a sub-G1 population at all comparisons. Numbers inside the columns indicate cell population distribution (%) at each cell-cycle phase. (TIF) [file pgen.1008808.s002.tif]

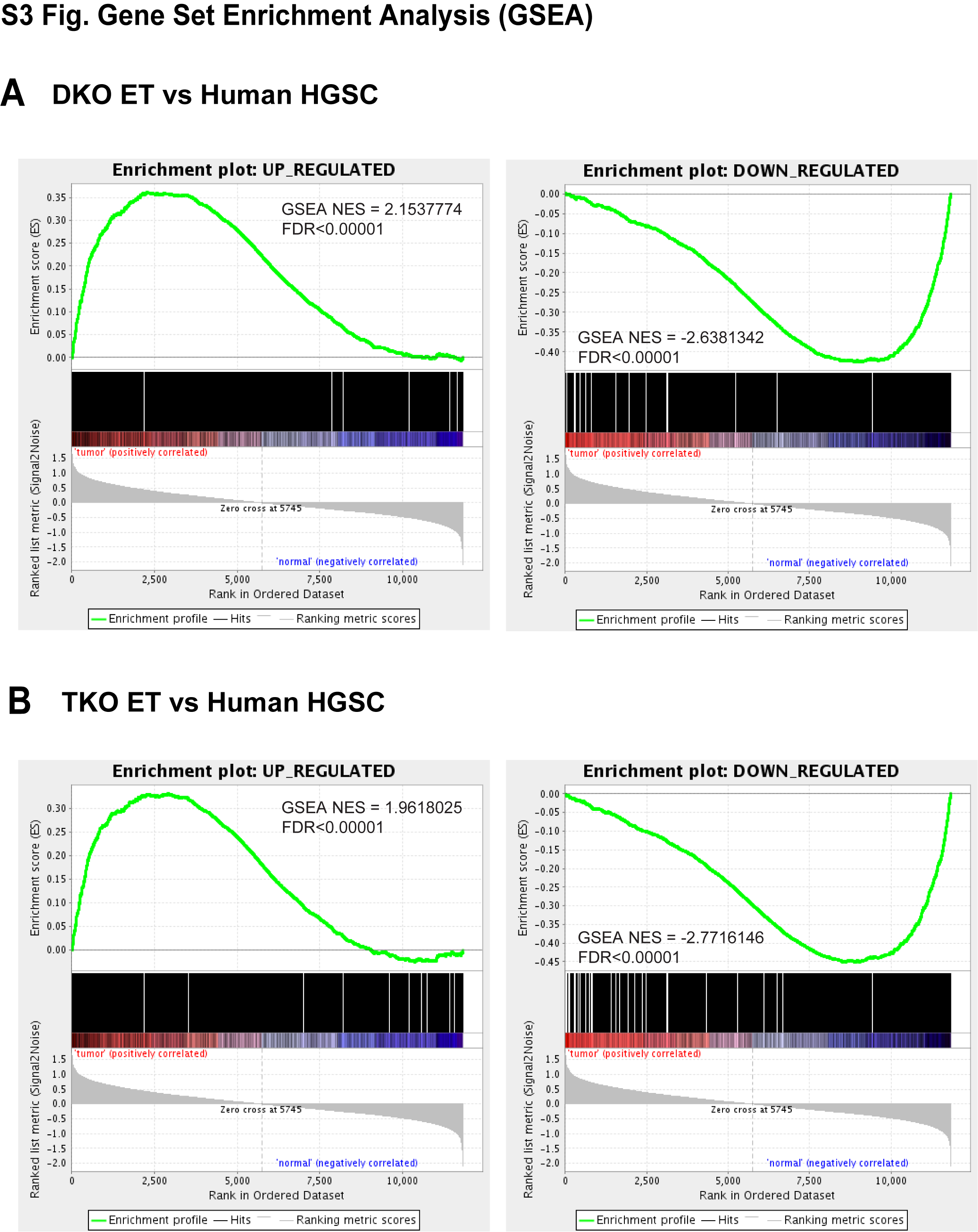

Supplement: S3 Fig — This analysis assesses similarity in gene expression between mouse (DKO and TKO) HGSCs and human HGSC from TCGA data [14]. Both DKO and TKO HGSCs (early-stage tumors, primary tumors, and metastatic tumors) were significantly enriched with the genes that are upregulated and downregulated in human HGSC. The representative enrichment plots indicate high significance of correlation between DKO ET and human HGSC (A) and between TKO ET and human HGSC (B) for the upregulated and downregulated genes. FDR<0.00001 (for all GSEAs between mouse HGSCs and human HGSC). ET, early-stage fallopian tube HGSC. NES, normalized enrichment score. FDR, false discovery rate. (TIF) [file pgen.1008808.s003.tif]
