# Supplementary material for: In vivo modeling of metastatic human high-grade serous ovarian cancer in mice
Source: PLoS Genet. 2020 Jun 4;16(6):e1008808. doi: 10.1371/journal.pgen.1008808 (PMC7297383; doi:10.1371/journal.pgen.1008808)
Supplement: S1 Table — (DOCX) [file pgen.1008808.s004.docx]

**S1 Table.** Chromosome gain and loss frequencies in human, mouse TKO, and DKO HGSCs.

| HGSC | | | TKO | | DKO | |
| --- | --- | --- | --- | --- | --- | --- |
| Human chr | **Gain frequency** | **Loss frequency** | **Gain frequency** | **Loss frequency** | **Gain frequency** | **Loss frequency** |
| 1q | 47%* |  | 3/12 (25%) |  | 1/12 (8%) |  |
| 3q | 59%* |  | 4/12 (33%) |  | 1/12 (8%) |  |
| 4p |  | 65%^†^ |  | 3/12 (25%) |  | 0/12 (0%) |
| 4q |  | 67%^†^ |  | 8/12 (67%) |  | 6/12 (50%) |
| 5q |  | 51%^†^ |  | 8/12 (67%) |  | 3/12 (25%) |
| 6p | 47%* |  | 1/12 (8%) |  | 1/12 (8%) |  |
| 6q |  | 59%^†^ |  | 6/12 (50%) |  | 11/12 (92%) |
| 7q | 47%* |  | 4/12 (33%) |  | 0/12 (0%) |  |
| 8p |  | 74%^†^ |  | 0/12 (0%) |  | 5/12 (42%) |
| 8q | 65%* | 44% | 5/12 (42%) |  | 1/12 (8%) |  |
| 9p |  | 66%^†^ |  | 6/12 (50%) |  | 11/12 (92%) |
| 9q |  | 60%^†^ |  | 6/12 (50%) |  | 11/12 (92%) |
| 11p |  | 49%^†^ |  | 3/12 (25%) |  | 1/12 (8%) |
| 11q |  | 41%^†^ |  | 4/12 (33%) |  | 2/12 (17%) |
| 12p | 54%* |  | 0/12 (0%) |  | 0/12 (0%) |  |
| 13q |  | 64%^†^ |  | 4/12 (33%) |  | 5/12 (42%) |
| 14q |  | 49%^†^ |  | 1/12 (8%) |  | 6/12 (50%) |
| 15q |  | 55%^†^ |  | 3/12 (25%) |  | 2/12 (17%) |
| 16p |  | 70%^†^ |  | 6/12 (50%) |  | 2/12 (17%) |
| 16q |  | 80%^†^ |  | 0/12 (0%) |  | 0/12 (0%) |
| 17p |  | 87%^†^ |  | 2/12 (17%) |  | 0/12 (0%) |
| 17q |  | 75%^†^ |  | 2/12 (17%) |  | 0/12 (0%) |
| 18p |  | 58%^†^ |  | 4/12 (33%) |  | 2/12 (17%) |
| 18q |  | 68%^†^ |  | 3/12 (25%) |  | 2/12 (17%) |
| 19p |  | 60%^†^ |  | 2/12 (17%) |  | 1/12 (8%) |
| 19q |  | 54%^†^ |  | 4/12 (33%) |  | 2/12 (17%) |
| 20p | 56%* |  | 4/12 (33%) |  | 0/12 (0%) |  |
| 20q | 61%* |  | 4/12 (33%) |  | 0/12 (0%) |  |
| 22q |  | 79%^†^ |  | 4/12 (33%) |  | 0/12 (0%) |

* Significant chromosome gain in human HGSC

^†^ Significant chromosome loss in human HGSC
