# Supplementary material for: In vivo modeling of metastatic human high-grade serous ovarian cancer in mice
Source: PLoS Genet. 2020 Jun 4;16(6):e1008808. doi: 10.1371/journal.pgen.1008808 (PMC7297383; doi:10.1371/journal.pgen.1008808)
Supplement: S2 Table — (DOCX) [file pgen.1008808.s005.docx]

|  |  |  |  |  |  |  |  |
| --- | --- | --- | --- | --- | --- | --- | --- |
|  | Host mouse | Number of injected cells (IP) | Tissue source | Total number of mice used | Tumor formation after injection | Rate of peritoneal metastasis | Rate of ascites development |
|  |  |  |  |  |  |  |  |
|  |  |  |  |  |  |  |  |
| TKO HGSC  Tissue cells | C57BL/129Sv | N/A | Primary Tumor | 2 | N/A | 0% (0/2) | 0% (0/2) |
|  |  | N/A | Metastatic tumor | 4 | 48 days  (1.6 months) | 75% (3/4) | 75% (3/4) |
|  |  | N/A | Ascites | 2 | 64 days  (2.1 months) | 50% (1/2) | 50% (1/2) |
|  |  | Total | | 8 |  | 50% (4/8) | 50% (4/8) |
|  |  |  |  |  |  |  |  |
| TKO HGSC  Cultured cells | DKO control  littermates  (*Dicer1* ^flox/flox^ *Pten* ^flox/flox^) | 4.9 x 10^6^ | Primary Tumor | 5 | 71 days  (2.4 months) | 20% (1/5) | 20% (1/5) |
|  |  | 5.6 x 10^6^ | Primary Tumor | 5 | 46 days  (1.5 months) | 20% (1/5) | 20% (1/5) |
|  |  | 3.2 x 10^6^ | Primary Tumor | 5 | 96 days ± 8.2 (3.2 months) | 80% (4/5) | 100% (5/5) |
|  |  | Total | | 15 |  | 40% (6/15) | 46.6% (7/15) |

**S2 Table**. *In vivo* transplantation of mouse HGSCs.

IP: Intraperitoneal injection
