# Supplementary material for: In vivo modeling of metastatic human high-grade serous ovarian cancer in mice
Source: PLoS Genet. 2020 Jun 4;16(6):e1008808. doi: 10.1371/journal.pgen.1008808 (PMC7297383; doi:10.1371/journal.pgen.1008808)
Supplement: S3 Table — (DOCX) [file pgen.1008808.s006.docx]

| Age  (month) | Tumor development (%) | | *P* value | Metastasis (%) | | *P* value |
| --- | --- | --- | --- | --- | --- | --- |
|  | DKO | TKO |  | DKO | TKO |  |
| 0 | 0 | 0 |  | 0 | 0 |  |
| 1 | 0 | 2.5 | 0.1531 | 0 | 0 |  |
| 2 | 0 | 7.1 | **0.0071** | 0 | 0 |  |
| 3 | 6.67 | 43.3 | **<0.0001** | 0 | 13.3 | **0.0002** |
| 4 | 23.1 | 84.4 | **<0.0001** | 7.7 | 28.1 | **0.0002** |
| 5 | 66.7 | 94.6 | **<0.0001** | 33.3 | 73 | **<0.0001** |
| 6 | 92.2 | 94.6 | 0.3895 | 47.1 | 94.6 | **<0.0001** |
| 7 | 93.8 | 100 | **0.0129** | 64.6 | 100 | **<0.0001** |
| 8 | 97.4 | 100 | 0.081 | 87.2 | 100 | **0.0002** |
| 9 | 97.9 | 100 | 0.1552 | 83.3 | 100 | **<0.0001** |
| >10 | 100 | 100 |  | 90.5 | 100 | **0.0021** |

**S3 Table.** Tumor development and metastasis in DKO and TKO mice.
