# Supplementary material for: In vivo modeling of metastatic human high-grade serous ovarian cancer in mice
Source: PLoS Genet. 2020 Jun 4;16(6):e1008808. doi: 10.1371/journal.pgen.1008808 (PMC7297383; doi:10.1371/journal.pgen.1008808)
Supplement: S4 Table — (DOCX) [file pgen.1008808.s007.docx]

**S4 Table.** Sequences of quantitative real-time PCR primers.

| Gene Name | Direction | Primer Sequence (5’-3’) |
| --- | --- | --- |
| Mouse *Rplp0* (36B4)  (Internal Control) | F | TGACATCGTCTTTAAACCCCG |
|  | R | TGTCTGCTCCCACAATGAAG |
| Mouse *Gapdh*  (Internal Control) | F | GTGCTGAGTATGTCGTGGAGTCTA |
|  | R | AAAGTTGTCATGGATGACCTTGG |
| Mouse *Dicer1* negative | F | TTCCTCCTGGTTATGTGGTAAACC |
|  | R | CACACTGCACCAAAACCAACACTATT |
| Mouse *Dicer1* | F | AAAGCAGAACTCTAATGCCCCGT |
|  | R | AGAGGTGCCTTTCGTTTAGGAAC |
| Mouse *Pten* negative | F | AGGGTCTCAGGGTCTCAGTGT |
|  | R | CTCAGTTCGCAGAGTGCTTGC |
| Mouse *Pten* | F | GTGAAGATGACAATCATGTTGCAGC |
|  | R | CCTGTTTCCCAATAAATTCTTGGTCC |
| Mouse *Pcna* | F | GGGTGAAGTTTTCTGCAAGTG |
|  | R | GTACCTCAGAGCAAACGTTAGG |
| Mouse *Brca1* | F | TCTGAAGACTGCTCGCAGAGTGATA |
|  | R | CCAGCACAGCTTCCAGGTGA |
| Mouse *Brca2* | F | GCCTCCACTTGCTGTGCTTC |
|  | R | TGCCATCTGGGCTGAGTGAG |
| Mouse *Fancd2* | F | AGCTGGCAAAGAAGTCTCACA |
|  | R | GTGTAGGACTCCAGGCCATT |
| Mouse *Foxm1* | F | GCCATGATACAGTTTGCCATC |
|  | R | AGAGAAAGGTTGTGACGAATAGAG |
| Mouse *Aurkb* | F | AGGGAGAACTGAAGATTGCAG |
|  | R | CCCGATGCACCATAGATCTAC |
| Mouse *Plk1* | F | TGTAGTTTTGGAGCTCTGTCG |
|  | R | TCCCTGTGAATGACCTGATTG |

F: Forward; R: Reverse
