# Supplementary material for: In vivo modeling of metastatic human high-grade serous ovarian cancer in mice
Source: PLoS Genet. 2020 Jun 4;16(6):e1008808. doi: 10.1371/journal.pgen.1008808 (PMC7297383; doi:10.1371/journal.pgen.1008808)
Supplement: S5 Table — (DOCX) [file pgen.1008808.s008.docx]

**S5 Table.** Human data information for CIN70 analysis and GSEA.

These human HGSC data were obtained from the TCGA dataset (Nature, 2011) at https://www.cbioportal.org/

| **TCGA main category** | **Sample type** | **Sample ID** |
| --- | --- | --- |
| TCGA Ovarian Serous Cystadenocarcinoma | Primary Tumor | TCGA-23-1109-01 |
| TCGA Ovarian Serous Cystadenocarcinoma | Primary Tumor | TCGA-04-1364-01 |
| TCGA Ovarian Serous Cystadenocarcinoma | Primary Tumor | TCGA-24-2019-01 |
| TCGA Ovarian Serous Cystadenocarcinoma | Primary Tumor | TCGA-13-1492-01 |
| TCGA Ovarian Serous Cystadenocarcinoma | Primary Tumor | TCGA-25-1322-01 |
| TCGA Ovarian Serous Cystadenocarcinoma | Primary Tumor | TCGA-04-1348-01 |
| TCGA Ovarian Serous Cystadenocarcinoma | Primary Tumor | TCGA-59-A5PD-01 |
| TCGA Ovarian Serous Cystadenocarcinoma | Primary Tumor | TCGA-10-0933-01 |
| TCGA Ovarian Serous Cystadenocarcinoma | Primary Tumor | TCGA-24-1552-01 |
| TCGA Ovarian Serous Cystadenocarcinoma | Primary Tumor | TCGA-09-1674-01 |
| TCGA Ovarian Serous Cystadenocarcinoma | Primary Tumor | TCGA-20-1683-01 |
| TCGA Ovarian Serous Cystadenocarcinoma | Primary Tumor | TCGA-24-1469-01 |
| TCGA Ovarian Serous Cystadenocarcinoma | Primary Tumor | TCGA-13-1506-01 |
| TCGA Ovarian Serous Cystadenocarcinoma | Primary Tumor | TCGA-24-1474-01 |
| TCGA Ovarian Serous Cystadenocarcinoma | Primary Tumor | TCGA-10-0938-01 |
| TCGA Ovarian Serous Cystadenocarcinoma | Primary Tumor | TCGA-13-A5FT-01 |
| TCGA Ovarian Serous Cystadenocarcinoma | Primary Tumor | TCGA-13-1410-01 |
| TCGA Ovarian Serous Cystadenocarcinoma | Primary Tumor | TCGA-25-1871-01 |
| TCGA Ovarian Serous Cystadenocarcinoma | Primary Tumor | TCGA-13-0893-01 |
| TCGA Ovarian Serous Cystadenocarcinoma | Primary Tumor | TCGA-13-0760-01 |
| TCGA Ovarian Serous Cystadenocarcinoma | Primary Tumor | TCGA-29-1698-01 |
| TCGA Ovarian Serous Cystadenocarcinoma | Primary Tumor | TCGA-24-0970-01 |
| TCGA Ovarian Serous Cystadenocarcinoma | Primary Tumor | TCGA-09-2053-01 |
| TCGA Ovarian Serous Cystadenocarcinoma | Primary Tumor | TCGA-24-2027-01 |
| TCGA Ovarian Serous Cystadenocarcinoma | Primary Tumor | TCGA-13-0885-01 |
| TCGA Ovarian Serous Cystadenocarcinoma | Primary Tumor | TCGA-24-1548-01 |
| TCGA Ovarian Serous Cystadenocarcinoma | Primary Tumor | TCGA-31-1950-01 |
| TCGA Ovarian Serous Cystadenocarcinoma | Primary Tumor | TCGA-23-2084-01 |
| TCGA Ovarian Serous Cystadenocarcinoma | Primary Tumor | TCGA-24-1463-01 |
| TCGA Ovarian Serous Cystadenocarcinoma | Primary Tumor | TCGA-20-1684-01 |
| TCGA Ovarian Serous Cystadenocarcinoma | Primary Tumor | TCGA-24-1424-01 |
| TCGA Ovarian Serous Cystadenocarcinoma | Primary Tumor | TCGA-61-1736-01 |
| TCGA Ovarian Serous Cystadenocarcinoma | Primary Tumor | TCGA-29-1697-01 |
| TCGA Ovarian Serous Cystadenocarcinoma | Primary Tumor | TCGA-57-1993-01 |
| TCGA Ovarian Serous Cystadenocarcinoma | Primary Tumor | TCGA-24-1842-01 |
| TCGA Ovarian Serous Cystadenocarcinoma | Primary Tumor | TCGA-30-1866-01 |
| TCGA Ovarian Serous Cystadenocarcinoma | Primary Tumor | TCGA-24-0982-01 |
| TCGA Ovarian Serous Cystadenocarcinoma | Primary Tumor | TCGA-23-1122-01 |
| TCGA Ovarian Serous Cystadenocarcinoma | Primary Tumor | TCGA-13-1488-01 |
| TCGA Ovarian Serous Cystadenocarcinoma | Primary Tumor | TCGA-13-1407-01 |
| TCGA Ovarian Serous Cystadenocarcinoma | Primary Tumor | TCGA-29-1762-01 |
| TCGA Ovarian Serous Cystadenocarcinoma | Primary Tumor | TCGA-24-2261-01 |
| TCGA Ovarian Serous Cystadenocarcinoma | Primary Tumor | TCGA-09-2044-01 |
| TCGA Ovarian Serous Cystadenocarcinoma | Primary Tumor | TCGA-36-1571-01 |
| TCGA Ovarian Serous Cystadenocarcinoma | Primary Tumor | TCGA-61-1741-01 |
| TCGA Ovarian Serous Cystadenocarcinoma | Primary Tumor | TCGA-23-1107-01 |
| TCGA Ovarian Serous Cystadenocarcinoma | Primary Tumor | TCGA-23-1809-01 |
| TCGA Ovarian Serous Cystadenocarcinoma | Primary Tumor | TCGA-13-1501-01 |
| TCGA Ovarian Serous Cystadenocarcinoma | Primary Tumor | TCGA-24-1556-01 |
| TCGA Ovarian Serous Cystadenocarcinoma | Primary Tumor | TCGA-24-1549-01 |
| TCGA Ovarian Serous Cystadenocarcinoma | Primary Tumor | TCGA-24-2020-01 |
| TCGA Ovarian Serous Cystadenocarcinoma | Primary Tumor | TCGA-04-1365-01 |
| TCGA Ovarian Serous Cystadenocarcinoma | Primary Tumor | TCGA-13-0906-01 |
| TCGA Ovarian Serous Cystadenocarcinoma | Primary Tumor | TCGA-61-1900-01 |
| TCGA Ovarian Serous Cystadenocarcinoma | Primary Tumor | TCGA-13-0799-01 |
| TCGA Ovarian Serous Cystadenocarcinoma | Primary Tumor | TCGA-24-1418-01 |
| TCGA Ovarian Serous Cystadenocarcinoma | Primary Tumor | TCGA-13-2060-01 |
| TCGA Ovarian Serous Cystadenocarcinoma | Primary Tumor | TCGA-36-1581-01 |
| TCGA Ovarian Serous Cystadenocarcinoma | Primary Tumor | TCGA-24-1550-01 |
| TCGA Ovarian Serous Cystadenocarcinoma | Primary Tumor | TCGA-04-1331-01 |
| TCGA Ovarian Serous Cystadenocarcinoma | Primary Tumor | TCGA-13-0727-01 |
| TCGA Ovarian Serous Cystadenocarcinoma | Primary Tumor | TCGA-25-1324-01 |
| TCGA Ovarian Serous Cystadenocarcinoma | Primary Tumor | TCGA-24-1843-01 |
| TCGA Ovarian Serous Cystadenocarcinoma | Primary Tumor | TCGA-29-1691-01 |
| TCGA Ovarian Serous Cystadenocarcinoma | Primary Tumor | TCGA-24-1928-01 |
| TCGA Ovarian Serous Cystadenocarcinoma | Primary Tumor | TCGA-30-1855-01 |
| TCGA Ovarian Serous Cystadenocarcinoma | Primary Tumor | TCGA-36-1576-01 |
| TCGA Ovarian Serous Cystadenocarcinoma | Primary Tumor | TCGA-23-1123-01 |
| TCGA Ovarian Serous Cystadenocarcinoma | Primary Tumor | TCGA-20-1685-01 |
| TCGA Ovarian Serous Cystadenocarcinoma | Primary Tumor | TCGA-25-2396-01 |
| TCGA Ovarian Serous Cystadenocarcinoma | Primary Tumor | TCGA-61-2098-01 |
| TCGA Ovarian Serous Cystadenocarcinoma | Primary Tumor | TCGA-25-1877-01 |
| TCGA Ovarian Serous Cystadenocarcinoma | Primary Tumor | TCGA-57-1582-01 |
| TCGA Ovarian Serous Cystadenocarcinoma | Primary Tumor | TCGA-36-1577-01 |
| TCGA Ovarian Serous Cystadenocarcinoma | Primary Tumor | TCGA-24-1426-01 |
| TCGA Ovarian Serous Cystadenocarcinoma | Primary Tumor | TCGA-23-2078-01 |
| TCGA Ovarian Serous Cystadenocarcinoma | Primary Tumor | TCGA-25-1320-01 |
| TCGA Ovarian Serous Cystadenocarcinoma | Primary Tumor | TCGA-24-1434-01 |
| TCGA Ovarian Serous Cystadenocarcinoma | Primary Tumor | TCGA-13-0730-01 |
| TCGA Ovarian Serous Cystadenocarcinoma | Primary Tumor | TCGA-25-1623-01 |
| TCGA Ovarian Serous Cystadenocarcinoma | Primary Tumor | TCGA-04-1530-01 |
| TCGA Ovarian Serous Cystadenocarcinoma | Primary Tumor | TCGA-25-1329-01 |
| TCGA Ovarian Serous Cystadenocarcinoma | Primary Tumor | TCGA-23-1110-01 |
| TCGA Ovarian Serous Cystadenocarcinoma | Primary Tumor | TCGA-29-1781-01 |
| TCGA Ovarian Serous Cystadenocarcinoma | Primary Tumor | TCGA-5X-AA5U-01 |
| TCGA Ovarian Serous Cystadenocarcinoma | Primary Tumor | TCGA-13-0920-01 |
| TCGA Ovarian Serous Cystadenocarcinoma | Primary Tumor | TCGA-13-1489-01 |
| TCGA Ovarian Serous Cystadenocarcinoma | Primary Tumor | TCGA-61-2113-01 |
| TCGA Ovarian Serous Cystadenocarcinoma | Primary Tumor | TCGA-24-1428-01 |
| TCGA Ovarian Serous Cystadenocarcinoma | Primary Tumor | TCGA-61-2102-01 |
| TCGA Ovarian Serous Cystadenocarcinoma | Primary Tumor | TCGA-59-2350-01 |
| TCGA Ovarian Serous Cystadenocarcinoma | Primary Tumor | TCGA-61-2012-01 |
| TCGA Ovarian Serous Cystadenocarcinoma | Primary Tumor | TCGA-13-0804-01 |
| TCGA Ovarian Serous Cystadenocarcinoma | Primary Tumor | TCGA-24-1417-01 |
| TCGA Ovarian Serous Cystadenocarcinoma | Primary Tumor | TCGA-24-0979-01 |
| TCGA Ovarian Serous Cystadenocarcinoma | Primary Tumor | TCGA-29-1695-01 |
| TCGA Ovarian Serous Cystadenocarcinoma | Primary Tumor | TCGA-59-2363-01 |
| TCGA Ovarian Serous Cystadenocarcinoma | Primary Tumor | TCGA-24-1436-01 |
| TCGA Ovarian Serous Cystadenocarcinoma | Primary Tumor | TCGA-29-1703-01 |
| TCGA Ovarian Serous Cystadenocarcinoma | Primary Tumor | TCGA-04-1341-01 |
| TCGA Ovarian Serous Cystadenocarcinoma | Primary Tumor | TCGA-10-0928-01 |
| TCGA Ovarian Serous Cystadenocarcinoma | Primary Tumor | TCGA-29-1705-01 |
| TCGA Ovarian Serous Cystadenocarcinoma | Primary Tumor | TCGA-23-1022-01 |
| TCGA Ovarian Serous Cystadenocarcinoma | Primary Tumor | TCGA-04-1517-01 |
| TCGA Ovarian Serous Cystadenocarcinoma | Primary Tumor | TCGA-04-1536-01 |
| TCGA Ovarian Serous Cystadenocarcinoma | Primary Tumor | TCGA-13-0886-01 |
| TCGA Ovarian Serous Cystadenocarcinoma | Primary Tumor | TCGA-61-1917-01 |
| TCGA Ovarian Serous Cystadenocarcinoma | Primary Tumor | TCGA-30-1718-01 |
| TCGA Ovarian Serous Cystadenocarcinoma | Primary Tumor | TCGA-23-1119-01 |
| TCGA Ovarian Serous Cystadenocarcinoma | Primary Tumor | TCGA-13-1512-01 |
| TCGA Ovarian Serous Cystadenocarcinoma | Primary Tumor | TCGA-23-1118-01 |
| TCGA Ovarian Serous Cystadenocarcinoma | Primary Tumor | TCGA-09-0364-01 |
| TCGA Ovarian Serous Cystadenocarcinoma | Primary Tumor | TCGA-29-A5NZ-01 |
| TCGA Ovarian Serous Cystadenocarcinoma | Primary Tumor | TCGA-61-1740-01 |
| TCGA Ovarian Serous Cystadenocarcinoma | Primary Tumor | TCGA-13-1509-01 |
| TCGA Ovarian Serous Cystadenocarcinoma | Primary Tumor | TCGA-13-0901-01 |
| TCGA Ovarian Serous Cystadenocarcinoma | Primary Tumor | TCGA-20-1682-01 |
| TCGA Ovarian Serous Cystadenocarcinoma | Primary Tumor | TCGA-25-2398-01 |
| TCGA Ovarian Serous Cystadenocarcinoma | Primary Tumor | TCGA-13-1511-01 |
| TCGA Ovarian Serous Cystadenocarcinoma | Primary Tumor | TCGA-29-1785-01 |
| TCGA Ovarian Serous Cystadenocarcinoma | Primary Tumor | TCGA-25-2393-01 |
| TCGA Ovarian Serous Cystadenocarcinoma | Primary Tumor | TCGA-13-0924-01 |
| TCGA Ovarian Serous Cystadenocarcinoma | Primary Tumor | TCGA-04-1347-01 |
| TCGA Ovarian Serous Cystadenocarcinoma | Primary Tumor | TCGA-09-1668-01 |
| TCGA Ovarian Serous Cystadenocarcinoma | Primary Tumor | TCGA-13-0905-01 |
| TCGA Ovarian Serous Cystadenocarcinoma | Primary Tumor | TCGA-09-2054-01 |
| TCGA Ovarian Serous Cystadenocarcinoma | Primary Tumor | TCGA-13-1405-01 |
| TCGA Ovarian Serous Cystadenocarcinoma | Primary Tumor | TCGA-31-1953-01 |
| TCGA Ovarian Serous Cystadenocarcinoma | Primary Tumor | TCGA-24-2281-01 |
| TCGA Ovarian Serous Cystadenocarcinoma | Primary Tumor | TCGA-29-1769-01 |
| TCGA Ovarian Serous Cystadenocarcinoma | Primary Tumor | TCGA-24-1603-01 |
| TCGA Ovarian Serous Cystadenocarcinoma | Primary Tumor | TCGA-13-1408-01 |
| TCGA Ovarian Serous Cystadenocarcinoma | Primary Tumor | TCGA-3P-A9WA-01 |
| TCGA Ovarian Serous Cystadenocarcinoma | Primary Tumor | TCGA-57-1583-01 |
| TCGA Ovarian Serous Cystadenocarcinoma | Primary Tumor | TCGA-24-1558-01 |
| TCGA Ovarian Serous Cystadenocarcinoma | Primary Tumor | TCGA-13-0923-01 |
| TCGA Ovarian Serous Cystadenocarcinoma | Primary Tumor | TCGA-13-1487-01 |
| TCGA Ovarian Serous Cystadenocarcinoma | Primary Tumor | TCGA-04-1338-01 |
| TCGA Ovarian Serous Cystadenocarcinoma | Primary Tumor | TCGA-13-1404-01 |
| TCGA Ovarian Serous Cystadenocarcinoma | Primary Tumor | TCGA-24-1105-01 |
| TCGA Ovarian Serous Cystadenocarcinoma | Primary Tumor | TCGA-09-1665-01 |
| TCGA Ovarian Serous Cystadenocarcinoma | Primary Tumor | TCGA-29-1701-01 |
| TCGA Ovarian Serous Cystadenocarcinoma | Primary Tumor | TCGA-24-1470-01 |
| TCGA Ovarian Serous Cystadenocarcinoma | Primary Tumor | TCGA-57-1994-01 |
| TCGA Ovarian Serous Cystadenocarcinoma | Primary Tumor | TCGA-31-1951-01 |
| TCGA Ovarian Serous Cystadenocarcinoma | Primary Tumor | TCGA-24-0975-01 |
| TCGA Ovarian Serous Cystadenocarcinoma | Primary Tumor | TCGA-13-1497-01 |
| TCGA Ovarian Serous Cystadenocarcinoma | Primary Tumor | TCGA-61-1995-01 |
| TCGA Ovarian Serous Cystadenocarcinoma | Primary Tumor | TCGA-23-1114-01 |
| TCGA Ovarian Serous Cystadenocarcinoma | Primary Tumor | TCGA-24-1924-01 |
| TCGA Ovarian Serous Cystadenocarcinoma | Primary Tumor | TCGA-24-0968-01 |
| TCGA Ovarian Serous Cystadenocarcinoma | Primary Tumor | TCGA-24-1430-01 |
| TCGA Ovarian Serous Cystadenocarcinoma | Primary Tumor | TCGA-24-2290-01 |
| TCGA Ovarian Serous Cystadenocarcinoma | Primary Tumor | TCGA-24-1431-01 |
| TCGA Ovarian Serous Cystadenocarcinoma | Primary Tumor | TCGA-25-2399-01 |
| TCGA Ovarian Serous Cystadenocarcinoma | Primary Tumor | TCGA-13-1499-01 |
| TCGA Ovarian Serous Cystadenocarcinoma | Primary Tumor | TCGA-09-2051-01 |
| TCGA Ovarian Serous Cystadenocarcinoma | Primary Tumor | TCGA-13-1481-01 |
| TCGA Ovarian Serous Cystadenocarcinoma | Primary Tumor | TCGA-29-1774-01 |
| TCGA Ovarian Serous Cystadenocarcinoma | Primary Tumor | TCGA-23-1029-01 |
| TCGA Ovarian Serous Cystadenocarcinoma | Primary Tumor | TCGA-04-1514-01 |
| TCGA Ovarian Serous Cystadenocarcinoma | Primary Tumor | TCGA-04-1356-01 |
| TCGA Ovarian Serous Cystadenocarcinoma | Primary Tumor | TCGA-13-0911-01 |
| TCGA Ovarian Serous Cystadenocarcinoma | Primary Tumor | TCGA-09-0369-01 |
| TCGA Ovarian Serous Cystadenocarcinoma | Primary Tumor | TCGA-13-0884-01 |
| TCGA Ovarian Serous Cystadenocarcinoma | Primary Tumor | TCGA-25-2400-01 |
| TCGA Ovarian Serous Cystadenocarcinoma | Primary Tumor | TCGA-25-1628-01 |
| TCGA Ovarian Serous Cystadenocarcinoma | Primary Tumor | TCGA-23-1032-01 |
| TCGA Ovarian Serous Cystadenocarcinoma | Primary Tumor | TCGA-61-2094-01 |
| TCGA Ovarian Serous Cystadenocarcinoma | Primary Tumor | TCGA-24-1850-01 |
| TCGA Ovarian Serous Cystadenocarcinoma | Primary Tumor | TCGA-23-1113-01 |
| TCGA Ovarian Serous Cystadenocarcinoma | Primary Tumor | TCGA-29-1702-01 |
| TCGA Ovarian Serous Cystadenocarcinoma | Primary Tumor | TCGA-13-1403-01 |
| TCGA Ovarian Serous Cystadenocarcinoma | Primary Tumor | TCGA-24-1845-01 |
| TCGA Ovarian Serous Cystadenocarcinoma | Primary Tumor | TCGA-25-2404-01 |
| TCGA Ovarian Serous Cystadenocarcinoma | Recurrent Tumor | TCGA-61-2008-02 |
| TCGA Ovarian Serous Cystadenocarcinoma | Primary Tumor | TCGA-29-1699-01 |
| TCGA Ovarian Serous Cystadenocarcinoma | Primary Tumor | TCGA-13-0890-01 |
| TCGA Ovarian Serous Cystadenocarcinoma | Primary Tumor | TCGA-13-0795-01 |
| TCGA Ovarian Serous Cystadenocarcinoma | Primary Tumor | TCGA-24-2297-01 |
| TCGA Ovarian Serous Cystadenocarcinoma | Primary Tumor | TCGA-24-1435-01 |
| TCGA Ovarian Serous Cystadenocarcinoma | Primary Tumor | TCGA-30-1860-01 |
| TCGA Ovarian Serous Cystadenocarcinoma | Primary Tumor | TCGA-24-2036-01 |
| TCGA Ovarian Serous Cystadenocarcinoma | Primary Tumor | TCGA-59-2348-01 |
| TCGA Ovarian Serous Cystadenocarcinoma | Primary Tumor | TCGA-25-1312-01 |
| TCGA Ovarian Serous Cystadenocarcinoma | Primary Tumor | TCGA-13-0899-01 |
| TCGA Ovarian Serous Cystadenocarcinoma | Primary Tumor | TCGA-24-2023-01 |
| TCGA Ovarian Serous Cystadenocarcinoma | Primary Tumor | TCGA-61-2109-01 |
| TCGA Ovarian Serous Cystadenocarcinoma | Primary Tumor | TCGA-25-1627-01 |
| TCGA Ovarian Serous Cystadenocarcinoma | Primary Tumor | TCGA-13-0887-01 |
| TCGA Ovarian Serous Cystadenocarcinoma | Primary Tumor | TCGA-61-1998-01 |
| TCGA Ovarian Serous Cystadenocarcinoma | Primary Tumor | TCGA-13-0900-01 |
| TCGA Ovarian Serous Cystadenocarcinoma | Primary Tumor | TCGA-13-1495-01 |
| TCGA Ovarian Serous Cystadenocarcinoma | Primary Tumor | TCGA-13-1498-01 |
| TCGA Ovarian Serous Cystadenocarcinoma | Primary Tumor | TCGA-61-2111-01 |
| TCGA Ovarian Serous Cystadenocarcinoma | Primary Tumor | TCGA-04-1651-01 |
| TCGA Ovarian Serous Cystadenocarcinoma | Primary Tumor | TCGA-61-1737-01 |
| TCGA Ovarian Serous Cystadenocarcinoma | Primary Tumor | TCGA-24-2026-01 |
| TCGA Ovarian Serous Cystadenocarcinoma | Primary Tumor | TCGA-59-2351-01 |
| TCGA Ovarian Serous Cystadenocarcinoma | Primary Tumor | TCGA-13-0891-01 |
| TCGA Ovarian Serous Cystadenocarcinoma | Primary Tumor | TCGA-24-1551-01 |
| TCGA Ovarian Serous Cystadenocarcinoma | Primary Tumor | TCGA-24-2293-01 |
| TCGA Ovarian Serous Cystadenocarcinoma | Primary Tumor | TCGA-04-1350-01 |
| TCGA Ovarian Serous Cystadenocarcinoma | Primary Tumor | TCGA-23-1030-01 |
| TCGA Ovarian Serous Cystadenocarcinoma | Primary Tumor | TCGA-24-1849-01 |
| TCGA Ovarian Serous Cystadenocarcinoma | Primary Tumor | TCGA-24-1419-01 |
| TCGA Ovarian Serous Cystadenocarcinoma | Primary Tumor | TCGA-24-1464-01 |
| TCGA Ovarian Serous Cystadenocarcinoma | Primary Tumor | TCGA-25-1323-01 |
| TCGA Ovarian Serous Cystadenocarcinoma | Primary Tumor | TCGA-04-1655-01 |
| TCGA Ovarian Serous Cystadenocarcinoma | Primary Tumor | TCGA-OY-A56P-01 |
| TCGA Ovarian Serous Cystadenocarcinoma | Primary Tumor | TCGA-36-1570-01 |
| TCGA Ovarian Serous Cystadenocarcinoma | Primary Tumor | TCGA-24-1544-01 |
| TCGA Ovarian Serous Cystadenocarcinoma | Primary Tumor | TCGA-29-1688-01 |
| TCGA Ovarian Serous Cystadenocarcinoma | Primary Tumor | TCGA-13-0725-01 |
| TCGA Ovarian Serous Cystadenocarcinoma | Primary Tumor | TCGA-09-1662-01 |
| TCGA Ovarian Serous Cystadenocarcinoma | Primary Tumor | TCGA-25-1635-01 |
| TCGA Ovarian Serous Cystadenocarcinoma | Primary Tumor | TCGA-61-2000-01 |
| TCGA Ovarian Serous Cystadenocarcinoma | Primary Tumor | TCGA-WR-A838-01 |
| TCGA Ovarian Serous Cystadenocarcinoma | Primary Tumor | TCGA-30-1861-01 |
| TCGA Ovarian Serous Cystadenocarcinoma | Primary Tumor | TCGA-24-1923-01 |
| TCGA Ovarian Serous Cystadenocarcinoma | Primary Tumor | TCGA-13-1496-01 |
| TCGA Ovarian Serous Cystadenocarcinoma | Primary Tumor | TCGA-24-1557-01 |
| TCGA Ovarian Serous Cystadenocarcinoma | Recurrent Tumor | TCGA-13-1489-02 |
| TCGA Ovarian Serous Cystadenocarcinoma | Primary Tumor | TCGA-25-1315-01 |
| TCGA Ovarian Serous Cystadenocarcinoma | Primary Tumor | TCGA-13-0800-01 |
| TCGA Ovarian Serous Cystadenocarcinoma | Primary Tumor | TCGA-13-1483-01 |
| TCGA Ovarian Serous Cystadenocarcinoma | Primary Tumor | TCGA-13-0801-01 |
| TCGA Ovarian Serous Cystadenocarcinoma | Primary Tumor | TCGA-13-0888-01 |
| TCGA Ovarian Serous Cystadenocarcinoma | Primary Tumor | TCGA-24-1427-01 |
| TCGA Ovarian Serous Cystadenocarcinoma | Primary Tumor | TCGA-25-1626-01 |
| TCGA Ovarian Serous Cystadenocarcinoma | Primary Tumor | TCGA-13-0762-01 |
| TCGA Ovarian Serous Cystadenocarcinoma | Primary Tumor | TCGA-29-1761-01 |
| TCGA Ovarian Serous Cystadenocarcinoma | Primary Tumor | TCGA-09-1669-01 |
| TCGA Ovarian Serous Cystadenocarcinoma | Primary Tumor | TCGA-36-1574-01 |
| TCGA Ovarian Serous Cystadenocarcinoma | Primary Tumor | TCGA-10-0926-01 |
| TCGA Ovarian Serous Cystadenocarcinoma | Primary Tumor | TCGA-25-1319-01 |
| TCGA Ovarian Serous Cystadenocarcinoma | Primary Tumor | TCGA-24-2288-01 |
| TCGA Ovarian Serous Cystadenocarcinoma | Primary Tumor | TCGA-29-1770-01 |
| TCGA Ovarian Serous Cystadenocarcinoma | Primary Tumor | TCGA-31-1959-01 |
| TCGA Ovarian Serous Cystadenocarcinoma | Primary Tumor | TCGA-25-2401-01 |
| TCGA Ovarian Serous Cystadenocarcinoma | Primary Tumor | TCGA-25-1314-01 |
| TCGA Ovarian Serous Cystadenocarcinoma | Recurrent Tumor | TCGA-29-1770-02 |
| TCGA Ovarian Serous Cystadenocarcinoma | Primary Tumor | TCGA-24-1423-01 |
| TCGA Ovarian Serous Cystadenocarcinoma | Primary Tumor | TCGA-25-1316-01 |
| TCGA Ovarian Serous Cystadenocarcinoma | Primary Tumor | TCGA-25-2391-01 |
| TCGA Ovarian Serous Cystadenocarcinoma | Primary Tumor | TCGA-23-1024-01 |
| TCGA Ovarian Serous Cystadenocarcinoma | Primary Tumor | TCGA-29-2414-01 |
| TCGA Ovarian Serous Cystadenocarcinoma | Primary Tumor | TCGA-04-1362-01 |
| TCGA Ovarian Serous Cystadenocarcinoma | Primary Tumor | TCGA-61-2003-01 |
| TCGA Ovarian Serous Cystadenocarcinoma | Primary Tumor | TCGA-13-0766-01 |
| TCGA Ovarian Serous Cystadenocarcinoma | Primary Tumor | TCGA-61-1919-01 |
| TCGA Ovarian Serous Cystadenocarcinoma | Primary Tumor | TCGA-20-0991-01 |
| TCGA Ovarian Serous Cystadenocarcinoma | Primary Tumor | TCGA-25-1321-01 |
| TCGA Ovarian Serous Cystadenocarcinoma | Primary Tumor | TCGA-61-1743-01 |
| TCGA Ovarian Serous Cystadenocarcinoma | Primary Tumor | TCGA-24-2298-01 |
| TCGA Ovarian Serous Cystadenocarcinoma | Primary Tumor | TCGA-09-1667-01 |
| TCGA Ovarian Serous Cystadenocarcinoma | Primary Tumor | TCGA-24-2033-01 |
| TCGA Ovarian Serous Cystadenocarcinoma | Primary Tumor | TCGA-23-1120-01 |
| TCGA Ovarian Serous Cystadenocarcinoma | Primary Tumor | TCGA-13-0897-01 |
| TCGA Ovarian Serous Cystadenocarcinoma | Primary Tumor | TCGA-23-1027-01 |
| TCGA Ovarian Serous Cystadenocarcinoma | Primary Tumor | TCGA-09-1673-01 |
| TCGA Ovarian Serous Cystadenocarcinoma | Primary Tumor | TCGA-04-1343-01 |
| TCGA Ovarian Serous Cystadenocarcinoma | Primary Tumor | TCGA-24-1846-01 |
| TCGA Ovarian Serous Cystadenocarcinoma | Primary Tumor | TCGA-24-2280-01 |
| TCGA Ovarian Serous Cystadenocarcinoma | Primary Tumor | TCGA-09-1670-01 |
| TCGA Ovarian Serous Cystadenocarcinoma | Primary Tumor | TCGA-VG-A8LO-01 |
| TCGA Ovarian Serous Cystadenocarcinoma | Primary Tumor | TCGA-25-1326-01 |
| TCGA Ovarian Serous Cystadenocarcinoma | Primary Tumor | TCGA-29-1763-01 |
| TCGA Ovarian Serous Cystadenocarcinoma | Primary Tumor | TCGA-10-0936-01 |
| TCGA Ovarian Serous Cystadenocarcinoma | Primary Tumor | TCGA-23-1028-01 |
| TCGA Ovarian Serous Cystadenocarcinoma | Recurrent Tumor | TCGA-29-2414-02 |
| TCGA Ovarian Serous Cystadenocarcinoma | Primary Tumor | TCGA-61-1738-01 |
| TCGA Ovarian Serous Cystadenocarcinoma | Primary Tumor | TCGA-25-1631-01 |
| TCGA Ovarian Serous Cystadenocarcinoma | Primary Tumor | TCGA-20-1686-01 |
| TCGA Ovarian Serous Cystadenocarcinoma | Primary Tumor | TCGA-36-1575-01 |
| TCGA Ovarian Serous Cystadenocarcinoma | Primary Tumor | TCGA-13-0724-01 |
| TCGA Ovarian Serous Cystadenocarcinoma | Primary Tumor | TCGA-24-1103-01 |
| TCGA Ovarian Serous Cystadenocarcinoma | Primary Tumor | TCGA-04-1332-01 |
| TCGA Ovarian Serous Cystadenocarcinoma | Primary Tumor | TCGA-13-1505-01 |
| TCGA Ovarian Serous Cystadenocarcinoma | Primary Tumor | TCGA-24-2262-01 |
| TCGA Ovarian Serous Cystadenocarcinoma | Primary Tumor | TCGA-24-1604-01 |
| TCGA Ovarian Serous Cystadenocarcinoma | Primary Tumor | TCGA-23-2081-01 |
| TCGA Ovarian Serous Cystadenocarcinoma | Primary Tumor | TCGA-24-1567-01 |
| TCGA Ovarian Serous Cystadenocarcinoma | Primary Tumor | TCGA-29-1766-01 |
| TCGA Ovarian Serous Cystadenocarcinoma | Primary Tumor | TCGA-09-1659-01 |
| TCGA Ovarian Serous Cystadenocarcinoma | Primary Tumor | TCGA-13-1411-01 |
| TCGA Ovarian Serous Cystadenocarcinoma | Primary Tumor | TCGA-13-1507-01 |
| TCGA Ovarian Serous Cystadenocarcinoma | Primary Tumor | TCGA-61-1728-01 |
| TCGA Ovarian Serous Cystadenocarcinoma | Primary Tumor | TCGA-04-1361-01 |
| TCGA Ovarian Serous Cystadenocarcinoma | Primary Tumor | TCGA-13-0797-01 |
| TCGA Ovarian Serous Cystadenocarcinoma | Primary Tumor | TCGA-10-0931-01 |
| TCGA Ovarian Serous Cystadenocarcinoma | Primary Tumor | TCGA-24-1562-01 |
| TCGA Ovarian Serous Cystadenocarcinoma | Primary Tumor | TCGA-61-1724-01 |
| TCGA Ovarian Serous Cystadenocarcinoma | Primary Tumor | TCGA-13-1482-01 |
| TCGA Ovarian Serous Cystadenocarcinoma | Primary Tumor | TCGA-25-1318-01 |
| TCGA Ovarian Serous Cystadenocarcinoma | Primary Tumor | TCGA-57-1585-01 |
| TCGA Ovarian Serous Cystadenocarcinoma | Primary Tumor | TCGA-25-1317-01 |
| TCGA Ovarian Serous Cystadenocarcinoma | Primary Tumor | TCGA-30-1714-01 |
| TCGA Ovarian Serous Cystadenocarcinoma | Recurrent Tumor | TCGA-29-1705-02 |
| TCGA Ovarian Serous Cystadenocarcinoma | Primary Tumor | TCGA-25-1634-01 |
| TCGA Ovarian Serous Cystadenocarcinoma | Primary Tumor | TCGA-09-2048-01 |
| TCGA Ovarian Serous Cystadenocarcinoma | Primary Tumor | TCGA-29-2425-01 |
| TCGA Ovarian Serous Cystadenocarcinoma | Primary Tumor | TCGA-10-0937-01 |
| TCGA Ovarian Serous Cystadenocarcinoma | Primary Tumor | TCGA-31-1944-01 |
| TCGA Ovarian Serous Cystadenocarcinoma | Primary Tumor | TCGA-24-1416-01 |
| TCGA Ovarian Serous Cystadenocarcinoma | Primary Tumor | TCGA-29-1784-01 |
| TCGA Ovarian Serous Cystadenocarcinoma | Primary Tumor | TCGA-30-1857-01 |
| TCGA Ovarian Serous Cystadenocarcinoma | Primary Tumor | TCGA-24-1555-01 |
| TCGA Ovarian Serous Cystadenocarcinoma | Primary Tumor | TCGA-61-2101-01 |
| TCGA Ovarian Serous Cystadenocarcinoma | Primary Tumor | TCGA-23-1116-01 |
| TCGA Ovarian Serous Cystadenocarcinoma | Primary Tumor | TCGA-24-1560-01 |
| TCGA Ovarian Serous Cystadenocarcinoma | Primary Tumor | TCGA-31-1946-01 |
| TCGA Ovarian Serous Cystadenocarcinoma | Primary Tumor | TCGA-24-1422-01 |
| TCGA Ovarian Serous Cystadenocarcinoma | Primary Tumor | TCGA-13-1510-01 |
| TCGA Ovarian Serous Cystadenocarcinoma | Recurrent Tumor | TCGA-23-1023-01 |
| TCGA Ovarian Serous Cystadenocarcinoma | Primary Tumor | TCGA-04-1337-01 |
| TCGA Ovarian Serous Cystadenocarcinoma | Primary Tumor | TCGA-61-1733-01 |
| TCGA Ovarian Serous Cystadenocarcinoma | Primary Tumor | TCGA-25-1630-01 |
| TCGA Ovarian Serous Cystadenocarcinoma | Primary Tumor | TCGA-61-2002-01 |
| TCGA Ovarian Serous Cystadenocarcinoma | Primary Tumor | TCGA-23-1111-01 |
| TCGA Ovarian Serous Cystadenocarcinoma | Primary Tumor | TCGA-59-2354-01 |
| TCGA Ovarian Serous Cystadenocarcinoma | Primary Tumor | TCGA-29-2428-01 |
| TCGA Ovarian Serous Cystadenocarcinoma | Primary Tumor | TCGA-61-2088-01 |
| TCGA Ovarian Serous Cystadenocarcinoma | Primary Tumor | TCGA-24-1425-01 |
| TCGA Ovarian Serous Cystadenocarcinoma | Primary Tumor | TCGA-29-1778-01 |
| TCGA Ovarian Serous Cystadenocarcinoma | Recurrent Tumor | TCGA-13-0913-02 |
| TCGA Ovarian Serous Cystadenocarcinoma | Primary Tumor | TCGA-24-1545-01 |
| TCGA Ovarian Serous Cystadenocarcinoma | Primary Tumor | TCGA-61-1725-01 |
| TCGA Ovarian Serous Cystadenocarcinoma | Primary Tumor | TCGA-09-1666-01 |
| TCGA Ovarian Serous Cystadenocarcinoma | Primary Tumor | TCGA-OY-A56Q-01 |
| TCGA Ovarian Serous Cystadenocarcinoma | Primary Tumor | TCGA-24-2254-01 |
| TCGA Ovarian Serous Cystadenocarcinoma | Primary Tumor | TCGA-24-1467-01 |
| TCGA Ovarian Serous Cystadenocarcinoma | Primary Tumor | TCGA-13-0883-01 |
| TCGA Ovarian Serous Cystadenocarcinoma | Primary Tumor | TCGA-61-2110-01 |
| TCGA Ovarian Serous Cystadenocarcinoma | Primary Tumor | TCGA-20-0987-01 |
| TCGA Ovarian Serous Cystadenocarcinoma | Primary Tumor | TCGA-24-1616-01 |
| TCGA Ovarian Serous Cystadenocarcinoma | Primary Tumor | TCGA-61-1911-01 |
| TCGA Ovarian Serous Cystadenocarcinoma | Primary Tumor | TCGA-24-2271-01 |
| TCGA Ovarian Serous Cystadenocarcinoma | Primary Tumor | TCGA-24-2267-01 |
| TCGA Ovarian Serous Cystadenocarcinoma | Primary Tumor | TCGA-10-0934-01 |
| TCGA Ovarian Serous Cystadenocarcinoma | Primary Tumor | TCGA-23-1026-01 |
| TCGA Ovarian Serous Cystadenocarcinoma | Primary Tumor | TCGA-10-0927-01 |
| TCGA Ovarian Serous Cystadenocarcinoma | Primary Tumor | TCGA-25-1632-01 |
| TCGA Ovarian Serous Cystadenocarcinoma | Primary Tumor | TCGA-04-1542-01 |
| TCGA Ovarian Serous Cystadenocarcinoma | Primary Tumor | TCGA-31-1956-01 |
| TCGA Ovarian Serous Cystadenocarcinoma | Primary Tumor | TCGA-24-1563-01 |
| TCGA Ovarian Serous Cystadenocarcinoma | Primary Tumor | TCGA-29-2427-01 |
| TCGA Ovarian Serous Cystadenocarcinoma | Primary Tumor | TCGA-61-1910-01 |
| TCGA Ovarian Serous Cystadenocarcinoma | Primary Tumor | TCGA-25-2392-01 |
| TCGA Ovarian Serous Cystadenocarcinoma | Primary Tumor | TCGA-13-0714-01 |
| TCGA Ovarian Serous Cystadenocarcinoma | Primary Tumor | TCGA-24-1413-01 |
| TCGA Ovarian Serous Cystadenocarcinoma | Primary Tumor | TCGA-13-0916-01 |
| TCGA Ovarian Serous Cystadenocarcinoma | Primary Tumor | TCGA-59-2352-01 |
| TCGA Ovarian Serous Cystadenocarcinoma | Primary Tumor | TCGA-61-2104-01 |
| TCGA Ovarian Serous Cystadenocarcinoma | Primary Tumor | TCGA-24-2024-01 |
| TCGA Ovarian Serous Cystadenocarcinoma | Recurrent Tumor | TCGA-29-1707-02 |
| TCGA Ovarian Serous Cystadenocarcinoma | Primary Tumor | TCGA-24-1564-01 |
| TCGA Ovarian Serous Cystadenocarcinoma | Primary Tumor | TCGA-30-1891-01 |
| TCGA Ovarian Serous Cystadenocarcinoma | Primary Tumor | TCGA-29-1694-01 |
| TCGA Ovarian Serous Cystadenocarcinoma | Primary Tumor | TCGA-29-1783-01 |
| TCGA Ovarian Serous Cystadenocarcinoma | Primary Tumor | TCGA-61-2009-01 |
| TCGA Ovarian Serous Cystadenocarcinoma | Primary Tumor | TCGA-29-1777-01 |
| TCGA Ovarian Serous Cystadenocarcinoma | Primary Tumor | TCGA-24-1844-01 |
| TCGA Ovarian Serous Cystadenocarcinoma | Primary Tumor | TCGA-13-1477-01 |
| TCGA Ovarian Serous Cystadenocarcinoma | Primary Tumor | TCGA-24-0966-01 |
| TCGA Ovarian Serous Cystadenocarcinoma | Primary Tumor | TCGA-59-2355-01 |
| TCGA Ovarian Serous Cystadenocarcinoma | Primary Tumor | TCGA-29-1710-01 |
| TCGA Ovarian Serous Cystadenocarcinoma | Primary Tumor | TCGA-61-1914-01 |
| TCGA Ovarian Serous Cystadenocarcinoma | Primary Tumor | TCGA-61-2016-01 |
| TCGA Ovarian Serous Cystadenocarcinoma | Primary Tumor | TCGA-61-2008-01 |
| TCGA Ovarian Serous Cystadenocarcinoma | Primary Tumor | TCGA-25-2042-01 |
| TCGA Ovarian Serous Cystadenocarcinoma | Primary Tumor | TCGA-13-1409-01 |
| TCGA Ovarian Serous Cystadenocarcinoma | Primary Tumor | TCGA-61-1918-01 |
| TCGA Ovarian Serous Cystadenocarcinoma | Primary Tumor | TCGA-13-0919-01 |
| TCGA Ovarian Serous Cystadenocarcinoma | Primary Tumor | TCGA-13-0908-01 |
| TCGA Ovarian Serous Cystadenocarcinoma | Primary Tumor | TCGA-23-2077-01 |
| TCGA Ovarian Serous Cystadenocarcinoma | Primary Tumor | TCGA-25-1313-01 |
| TCGA Ovarian Serous Cystadenocarcinoma | Primary Tumor | TCGA-13-0768-01 |
| TCGA Ovarian Serous Cystadenocarcinoma | Primary Tumor | TCGA-25-2409-01 |
| TCGA Ovarian Serous Cystadenocarcinoma | Primary Tumor | TCGA-13-0726-01 |
| TCGA Ovarian Serous Cystadenocarcinoma | Primary Tumor | TCGA-61-2092-01 |
| TCGA Ovarian Serous Cystadenocarcinoma | Primary Tumor | TCGA-25-1625-01 |
| TCGA Ovarian Serous Cystadenocarcinoma | Primary Tumor | TCGA-30-1892-01 |
| TCGA Ovarian Serous Cystadenocarcinoma | Primary Tumor | TCGA-24-1930-01 |
| TCGA Ovarian Serous Cystadenocarcinoma | Primary Tumor | TCGA-57-1584-01 |
| TCGA Ovarian Serous Cystadenocarcinoma | Primary Tumor | TCGA-24-2035-01 |
| TCGA Ovarian Serous Cystadenocarcinoma | Primary Tumor | TCGA-13-1485-01 |
| TCGA Ovarian Serous Cystadenocarcinoma | Primary Tumor | TCGA-36-1569-01 |
| TCGA Ovarian Serous Cystadenocarcinoma | Primary Tumor | TCGA-57-1586-01 |
| TCGA Ovarian Serous Cystadenocarcinoma | Primary Tumor | TCGA-09-2056-01 |
| TCGA Ovarian Serous Cystadenocarcinoma | Primary Tumor | TCGA-25-2397-01 |
| TCGA Ovarian Serous Cystadenocarcinoma | Primary Tumor | TCGA-09-0367-01 |
| TCGA Ovarian Serous Cystadenocarcinoma | Primary Tumor | TCGA-04-1519-01 |
| TCGA Ovarian Serous Cystadenocarcinoma | Primary Tumor | TCGA-61-1907-01 |
| TCGA Ovarian Serous Cystadenocarcinoma | Primary Tumor | TCGA-09-1661-01 |
| TCGA Ovarian Serous Cystadenocarcinoma | Primary Tumor | TCGA-25-1633-01 |
| TCGA Ovarian Serous Cystadenocarcinoma | Primary Tumor | TCGA-29-1776-01 |
| TCGA Ovarian Serous Cystadenocarcinoma | Primary Tumor | TCGA-29-1696-01 |
| TCGA Ovarian Serous Cystadenocarcinoma | Primary Tumor | TCGA-61-2097-01 |
| TCGA Ovarian Serous Cystadenocarcinoma | Primary Tumor | TCGA-36-1580-01 |
| TCGA Ovarian Serous Cystadenocarcinoma | Primary Tumor | TCGA-09-0366-01 |
| TCGA Ovarian Serous Cystadenocarcinoma | Primary Tumor | TCGA-04-1357-01 |
| TCGA Ovarian Serous Cystadenocarcinoma | Primary Tumor | TCGA-13-0720-01 |
| TCGA Ovarian Serous Cystadenocarcinoma | Primary Tumor | TCGA-23-1021-01 |
| TCGA Ovarian Serous Cystadenocarcinoma | Primary Tumor | TCGA-36-1578-01 |
| TCGA Ovarian Serous Cystadenocarcinoma | Primary Tumor | TCGA-29-1768-01 |
| TCGA Ovarian Serous Cystadenocarcinoma | Primary Tumor | TCGA-30-1853-01 |
| TCGA Ovarian Serous Cystadenocarcinoma | Primary Tumor | TCGA-24-2289-01 |
| TCGA Ovarian Serous Cystadenocarcinoma | Primary Tumor | TCGA-24-1104-01 |
| TCGA Ovarian Serous Cystadenocarcinoma | Primary Tumor | TCGA-61-2095-01 |
| TCGA Ovarian Serous Cystadenocarcinoma | Primary Tumor | TCGA-36-1568-01 |
| TCGA Ovarian Serous Cystadenocarcinoma | Primary Tumor | TCGA-24-1553-01 |
| TCGA Ovarian Serous Cystadenocarcinoma | Primary Tumor | TCGA-29-1693-01 |
| TCGA Ovarian Serous Cystadenocarcinoma | Primary Tumor | TCGA-24-1471-01 |
| TCGA Ovarian Serous Cystadenocarcinoma | Primary Tumor | TCGA-24-1565-01 |
| TCGA Ovarian Serous Cystadenocarcinoma | Primary Tumor | TCGA-13-0765-01 |
| TCGA Ovarian Serous Cystadenocarcinoma | Primary Tumor | TCGA-20-1687-01 |
| TCGA Ovarian Serous Cystadenocarcinoma | Primary Tumor | TCGA-24-1546-01 |
| TCGA Ovarian Serous Cystadenocarcinoma | Primary Tumor | TCGA-29-1711-01 |
| TCGA Ovarian Serous Cystadenocarcinoma | Primary Tumor | TCGA-30-1862-01 |
| TCGA Ovarian Serous Cystadenocarcinoma | Primary Tumor | TCGA-04-1648-01 |
| TCGA Ovarian Serous Cystadenocarcinoma | Primary Tumor | TCGA-09-2045-01 |
| TCGA Ovarian Serous Cystadenocarcinoma | Primary Tumor | TCGA-29-1690-01 |
| TCGA Ovarian Serous Cystadenocarcinoma | Primary Tumor | TCGA-25-1328-01 |
| TCGA Ovarian Serous Cystadenocarcinoma | Primary Tumor | TCGA-61-1721-01 |
| TCGA Ovarian Serous Cystadenocarcinoma | Primary Tumor | TCGA-24-2038-01 |

These human fallopian tube data were extracted from Genotype-Tissue Expression (GTEx) Project at https://www.gtexportal.org/home/

| **Category** | **Sample type** | **Sample ID** |
| --- | --- | --- |
| GTEX Fallopian Tube | Fallopian Tube | GTEX-S32W-1326-SM-4AD5Q |
| GTEX Fallopian Tube | Fallopian Tube | GTEX-T5JW-0326-SM-4DM6J |
| GTEX Fallopian Tube | Fallopian Tube | GTEX-U3ZN-1126-SM-4DXUL |
| GTEX Fallopian Tube | Fallopian Tube | GTEX-S341-0826-SM-4AD73 |
| GTEX Fallopian Tube | Fallopian Tube | GTEX-T6MO-1026-SM-4DM72 |
